# A Kernelisation Approach for Multiple *d*-Hitting Set and Its Application in Optimal Multi-Drug Therapeutic Combinations

**DOI:** 10.1371/journal.pone.0013055

**Published:** 2010-10-18

**Authors:** Drew Mellor, Elena Prieto, Luke Mathieson, Pablo Moscato

**Affiliations:** 1 Centre for Bioinformatics, Biomarker Discovery and Information Based Medicine, The University of Newcastle, Newcastle, Australia; 2 Information Based Medicine Program, Hunter Medical Research Institute, Newcastle, Australia; Hungarian Academy of Sciences, Hungary

## Abstract

Therapies consisting of a combination of agents are an attractive proposition, especially in the context of diseases such as cancer, which can manifest with a variety of tumor types in a single case. However uncovering usable drug combinations is expensive both financially and temporally. By employing computational methods to identify candidate combinations with a greater likelihood of success we can avoid these problems, even when the amount of data is prohibitively large. Hitting Set is a combinatorial problem that has useful application across many fields, however as it is *NP*-complete it is traditionally considered hard to solve exactly. We introduce a more general version of the problem (*α,β,d*)-Hitting Set, which allows more precise control over how and what the hitting set targets. Employing the framework of Parameterized Complexity we show that despite being *NP*-complete, the (*α,β,d*)-Hitting Set problem is fixed-parameter tractable with a kernel of size *O*(α*dk^d^*) when we parameterize by the size *k* of the hitting set and the maximum number α of the minimum number of hits, and taking the maximum degree *d* of the target sets as a constant. We demonstrate the application of this problem to multiple drug selection for cancer therapy, showing the flexibility of the problem in tailoring such drug sets. The fixed-parameter tractability result indicates that for low values of the parameters the problem can be solved quickly using exact methods. We also demonstrate that the problem is indeed practical, with computation times on the order of 5 seconds, as compared to previous Hitting Set applications using the same dataset which exhibited times on the order of 1 day, even with relatively relaxed notions for what constitutes a low value for the parameters. Furthermore the existence of a kernelization for (*α,β,d*)-Hitting Set indicates that the problem is readily scalable to large datasets.

## Introduction

Typically the selection of a drug therapy for a disease is limited to a single drug, however diseases such as cancer may present as a heterogeneous mix of subtypes of the general disease. In cases such as these multi-drug therapies may prove more effective than single drug therapies, and many trials have been conducted to this end [1]–[3]. Furthermore combinations of drugs may allow a more targeted approach for a selection of subtypes of a disease, while minimizing effects on unaffected cells. Unfortunately with the abundance of compounds available for the treatment of many conditions of interest, the time and expense in testing even all two drug combinations may be prohibitive. Therefore a smarter approach is needed. Vazquez [4] introduces the Hitting Set problem for this task in the context of oncological drug therapy. The Hitting Set problem is a combinatorial problem that proves extremely useful in modeling a large variety of problems in many domains including protein network discovery [5], metabolic network analysis [6], diagnostics [7]–[9], gene ontology [10] and gene expression analysis [11], [12].

### The Hitting Set Problem

Hitting Set is a combinatorial problem that models the problem of selecting a small group of elements to represent or cover a collection of sets. Such a group that covers every set in the collection is called a hitting set. Finding such a set without any constraint is simple, however if we required that the size of the hitting set be relatively small, the problem becomes computationally challenging (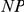-complete in a formal sense). This difficulty in obtaining solutions with desirable qualities thus requires more thoughtful approaches.

We now give some technical details and formal definitions of the problems of interest.

Hitting Set is equivalent to the Set Cover problem [13], and when otherwise unrestricted, is equivalent to the Red/Blue Dominating Set [14] problem and is related to the 
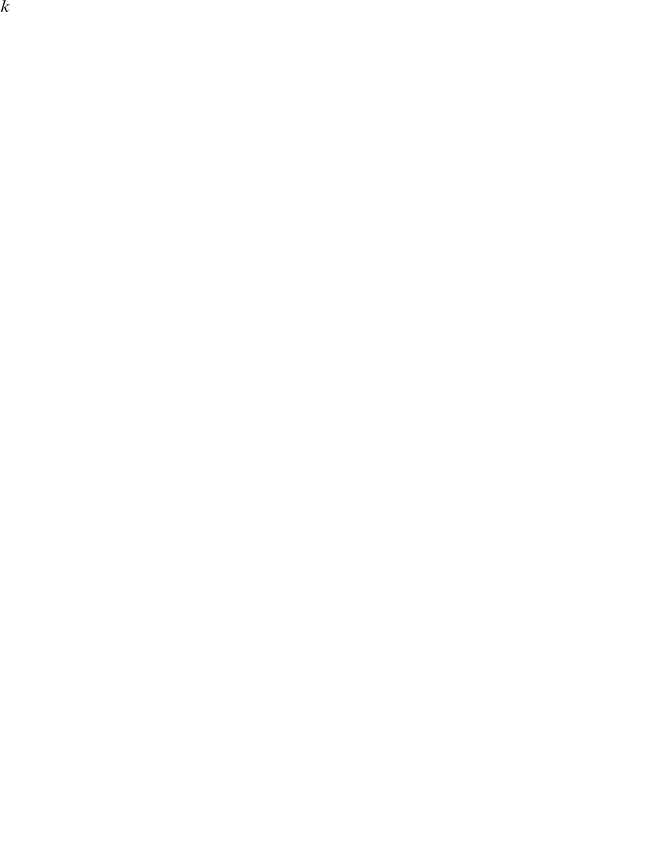
-Feature Set [15] problem.

The decision version of the Hitting Set problem is defined as follows:

Hitting Set

*Instance:* A set 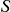 and a collection 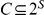 and an integer 
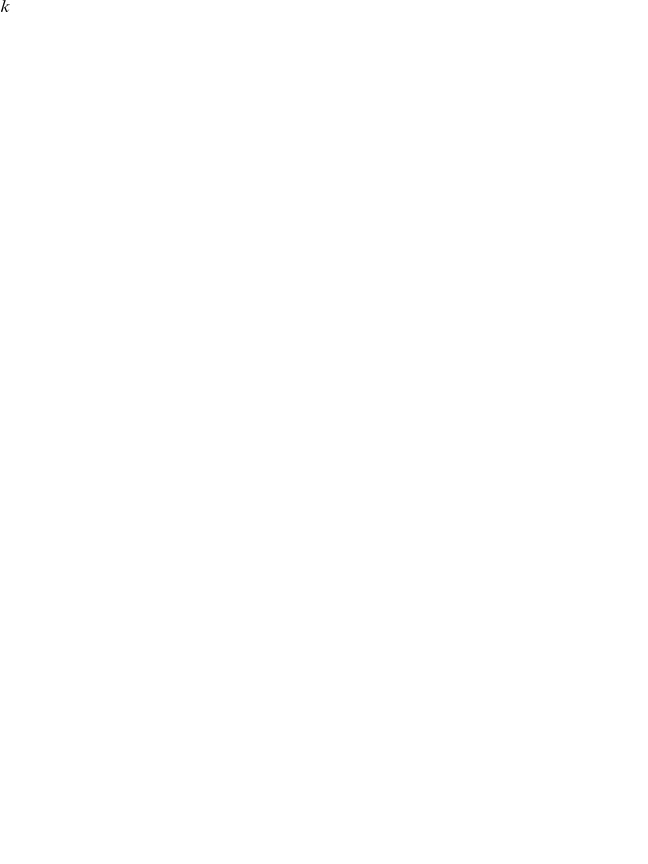
.

*Question:* Is there a set 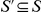 with 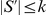 such that for every 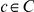 we have 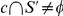
?

The set 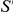 is called a *hitting set for* 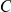, or simply a *hitting set*. For an element 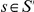 and an element 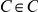 if 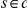 we say that 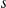 *hits* 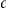. This problem is 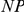-complete even when the maximum size of each element of 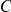 is two (by equivalence with Vertex Cover [13]) and 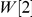-complete for parameter 
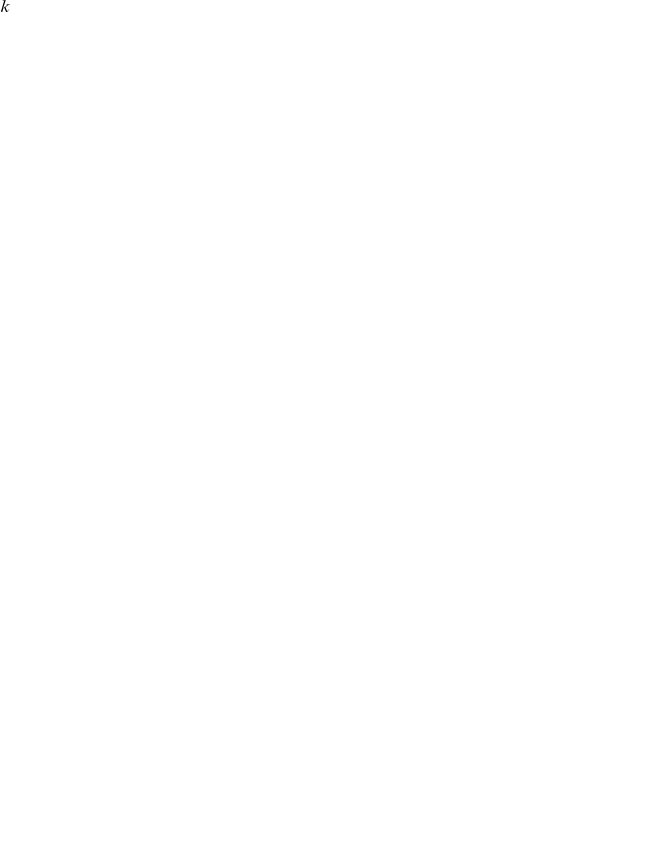
; Cotta and Moscato [16] give a parameterized proof via 
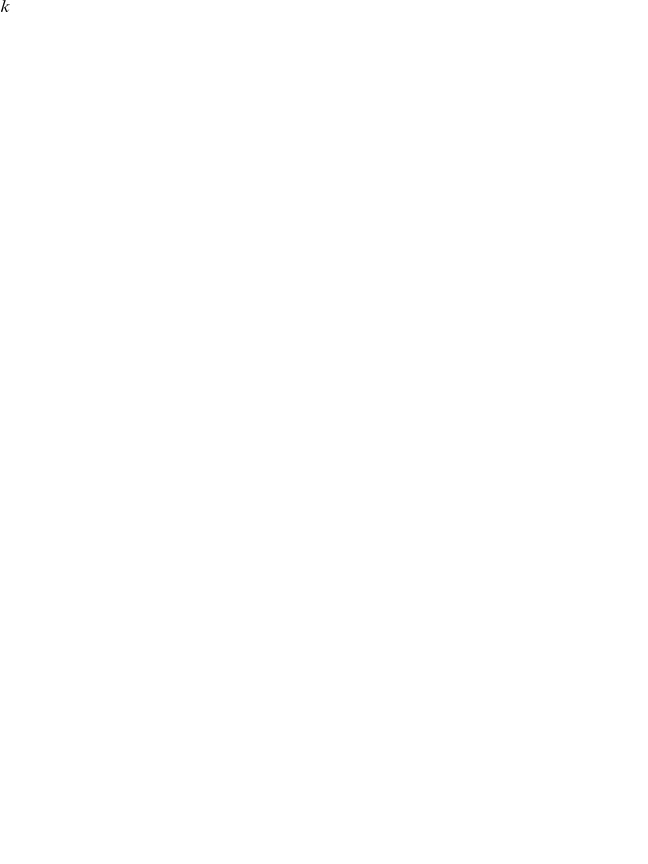
-Feature Set and Paz and Moran [17] give a proof which along with the equivalence of Hitting Set and Set Cover leads to the same result, though predates the parameterized complexity framework. However if we restrict the cardinality of the elements of 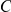 to 
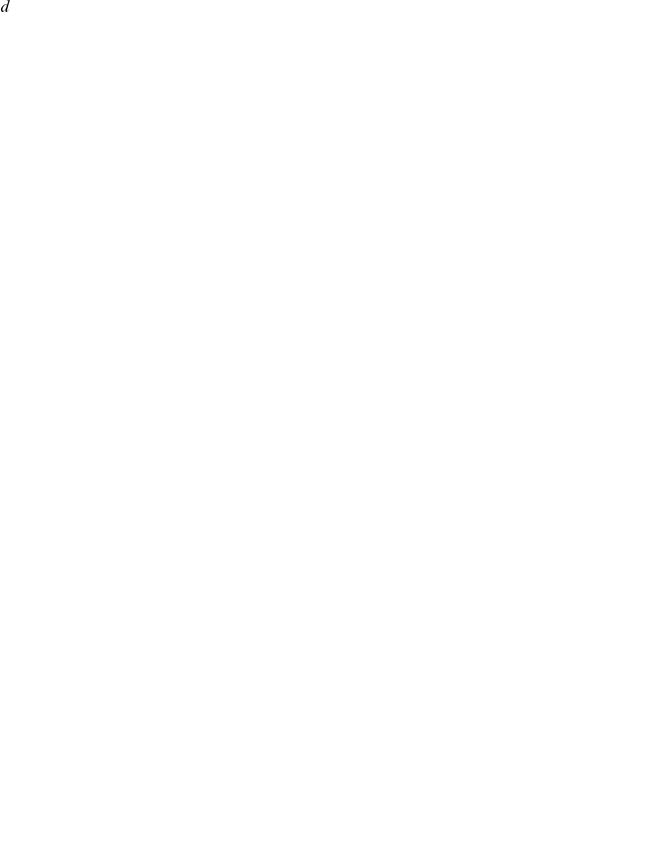
 the problem, while remaining 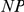-complete, becomes fixed-parameter tractable where 
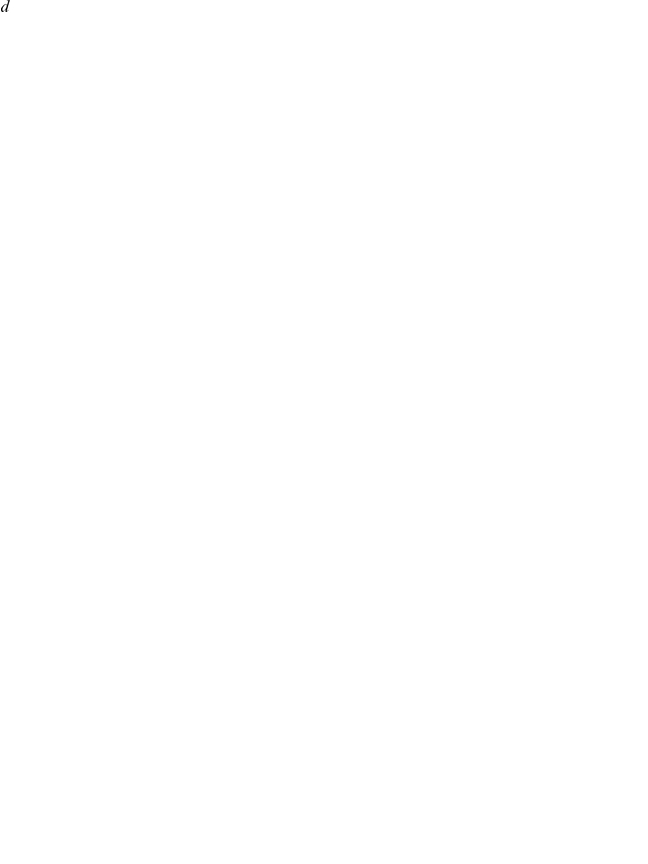
 is a constant and the parameter is 
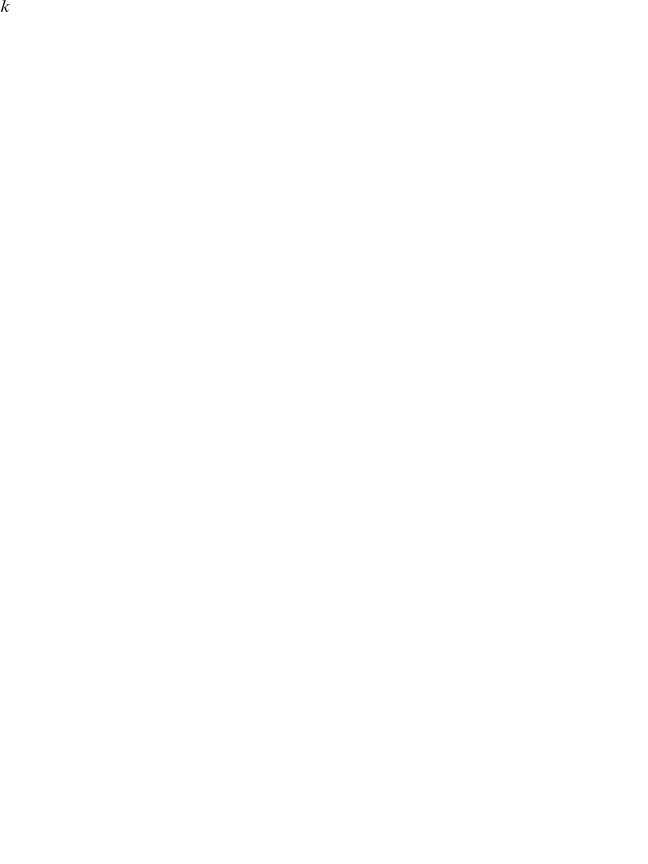
 [18]. In this case the problem is known as the Hitting Set for Sets of Size 
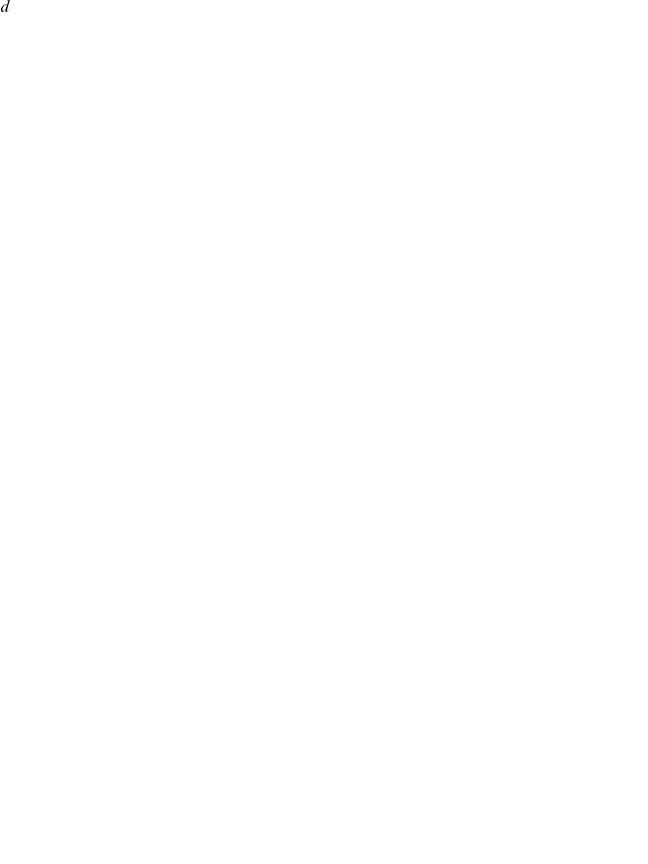
 or 
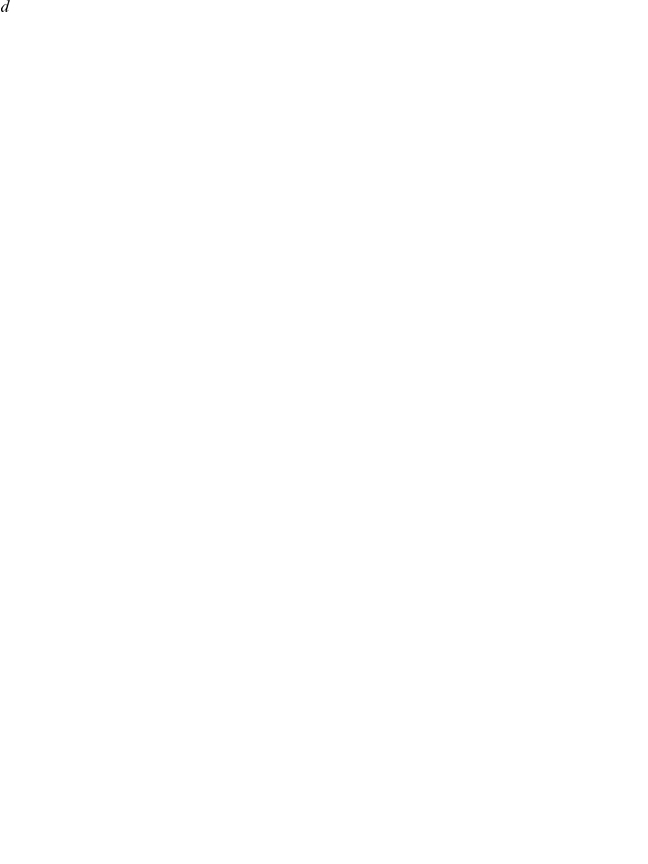
-Hitting Set problem. We note that Hitting Set has several equivalent formulations, in particular we choose to use the bipartite graph representation where 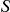 and 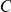 form the two partite vertex sets of the graph and an edge 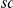 corresponds to the element 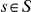 being an element of 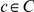. This allows us to employ some simplifying graph theoretic terminology and techniques. We generalize this problem to include the case where we may want the elements of 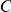 to be hit more than once. In particular this includes the case where we ask if all the sets of 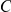 can be hit 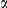 times, but extends to the case where the elements of 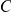 can be hit up to 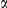 times. We encode this by the use of a hitting function 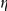. Our problem then becomes the 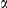-Multiple 
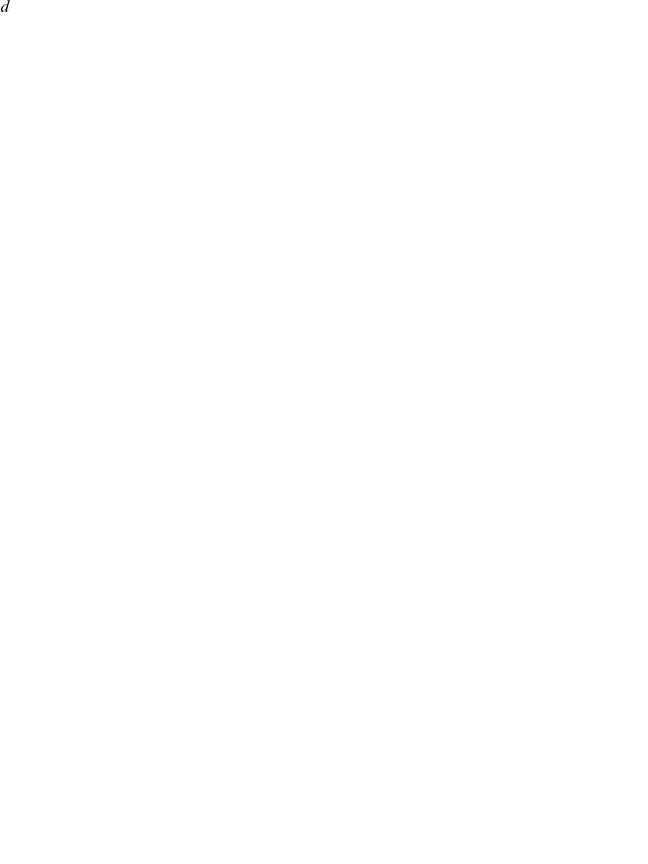
-Hitting Set (or (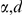)-Hitting Set):

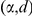
-Hitting Set

*Instance:* A bipartite graph 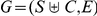 where for all 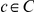 we have 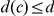, a hitting function 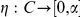 and an integer 
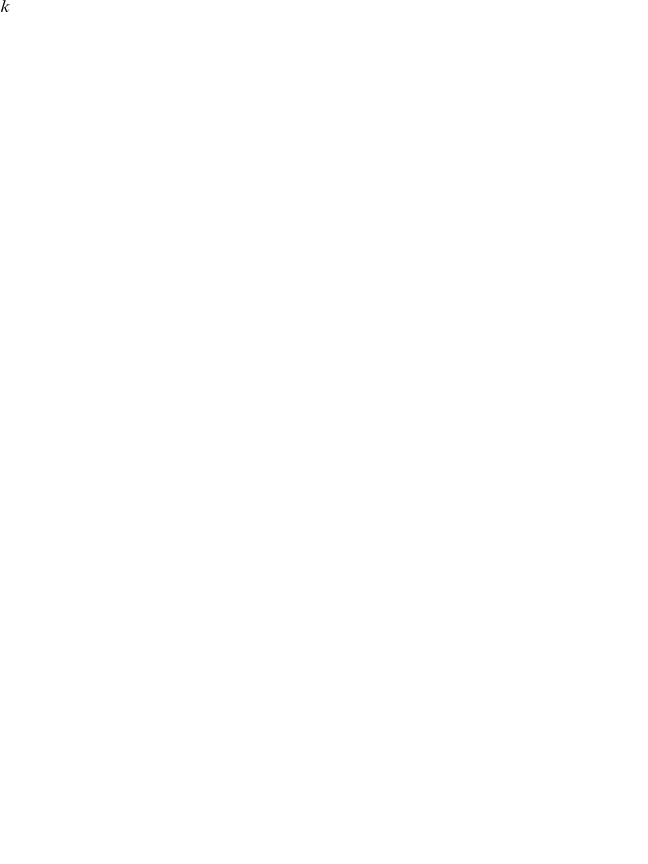
.

*Question:* Is there a set 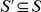 with 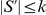 such that for every 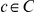 we have 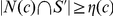
?

When 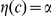 for all 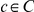, (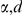)-Hitting Set can be 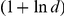-approximated in time 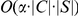 [19], but cannot be approximated with a factor of 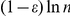 for any 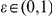 unless 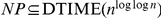 [20].

## Results and Discussion

### The Fixed-Parameter Tractability of (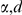)-Hitting Set

As we prove in the *Materials and Methods* section, the (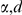)-Hitting Set problem is fixed-parameter tractable, and indeed a more general variant the (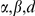)-Hitting Set problem is also fixed parameter tractable when we take the maximum degree 
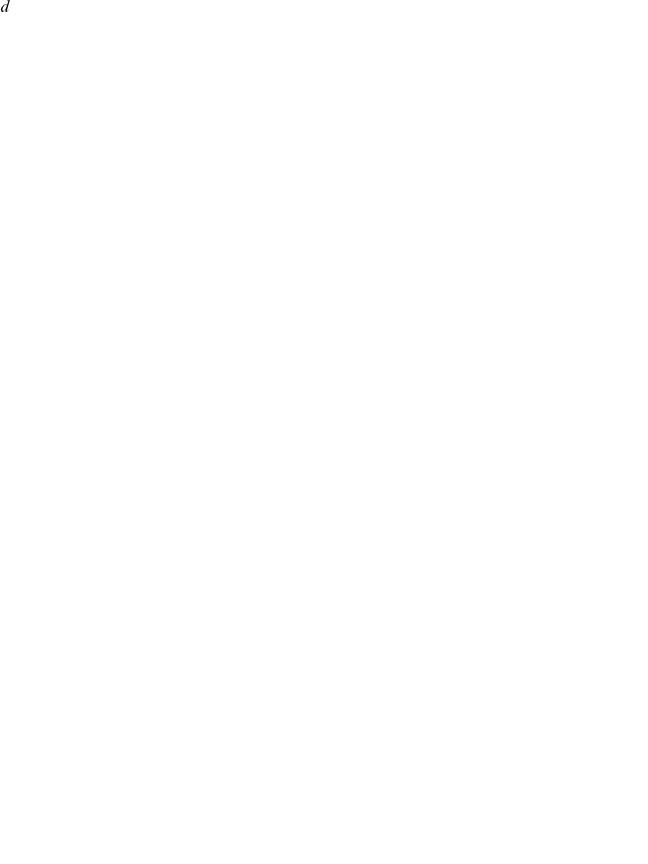
 of the class vertices 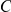 as a constant and the size 
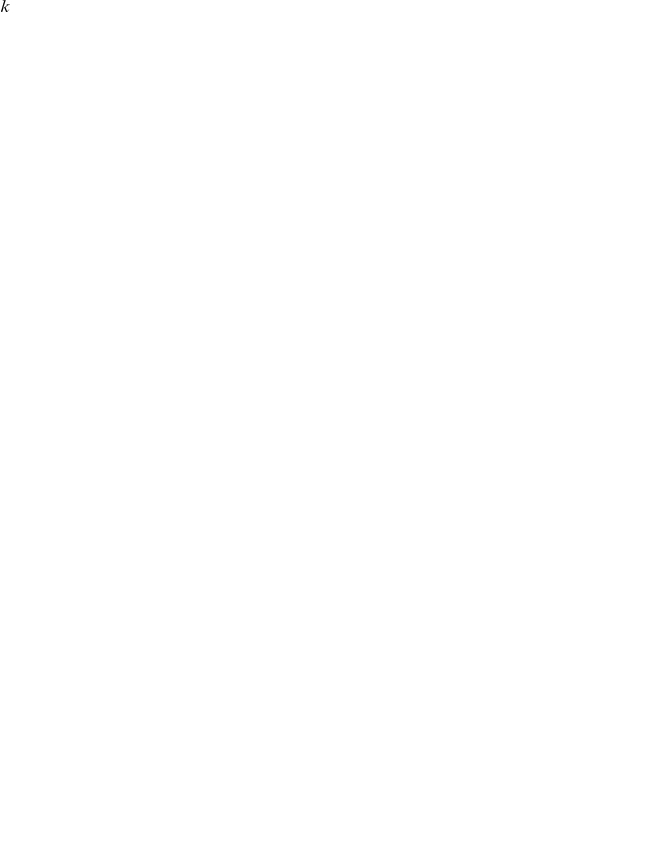
 of the hitting set and the maximum desired coverage 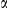 as a joint parameter. Though the problem is formally hard - which would normally give the intuition that an exact solution would be too expensive to compute - the fixed-parameter tractability indicates that it is likely that we can obtain an exact solution efficiently. Armed with this knowledge we proceed with the experiments of the following section, where we use the drug response data of the NCI60 anti-tumor drug screening program to determine a sets of drugs that hit cancerous cell lines multiple times. These drug sets are than mathematically supportable candidates for combination chemotherapies. Moreover we are able to tune the nature of the hitting sets via the numbers 
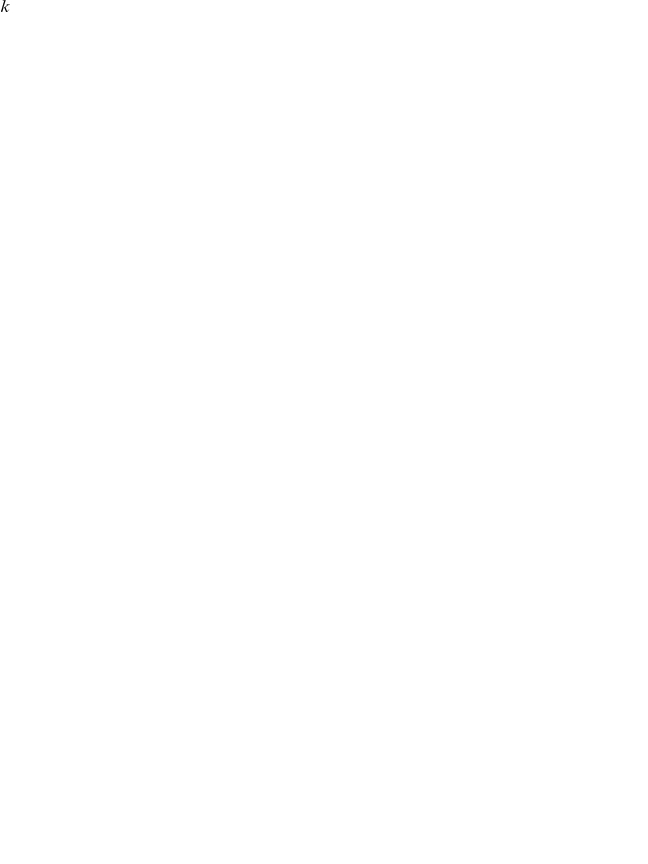
, 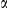 and 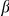, which allows us to control which cell lines are targetted (and which are specifically not) and how much each cell line is hit in the solution.

### A Comparative Application

The NCI60 human tumor anti-cancer drug screen dataset [21] was established in the 1980s as an enabling tool for anti-cancer drug development. Included in this dataset is response data for over 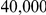 drugs against the 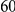 cell lines of the dataset. Vazquez [4] highlights the utility of a hitting set approach in developing multi-drug therapies for heterogeneous malignancies; given the plethora of available compounds, testing multi-drug combinations exhaustively is prohibitive if not impossible. Applying hitting set to efficacy data measured on an individual basis for each compound allows us to determine possible drug combinations that would provide the best chance of efficacy against many cancer types. Using the GI50 response NCI60 dataset (available from the DTP website [22]) Vazquez uncovers a minimum hitting set with three compounds that cumulatively gives a good response with all cell lines in the dataset, where a response is considered good if it is more than two standard deviations above the mean of the z-transformed response data. Vazquez uses first a greedy highest-degree-first approach to give an estimate of the maximum size of a minimum hitting set, followed by either an exhaustive search or simulated annealing, depending on the size of the hitting set. Vazquez reports times for such approaches on the order of one day on a desktop computer.

We revisit Vasquez's experiment, using data reduction (though it is not necessary to employ the more complex rules given in the kernelization proof) with IBM ILOG CPLEX [23] as the kernel solver by framing the problem as a integer programming problem. We use the same threshold for the z-transformation to identify significant response levels. Using this approach we reduce the time to solve the instance to less than 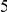 seconds, where most of the time is spent loading and reducing the data, with CPLEX solving the integer programming instance in approximately 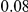 milliseconds. Furthermore this approach guarantees optimality in the size of the hitting set.

**Figure 1:**
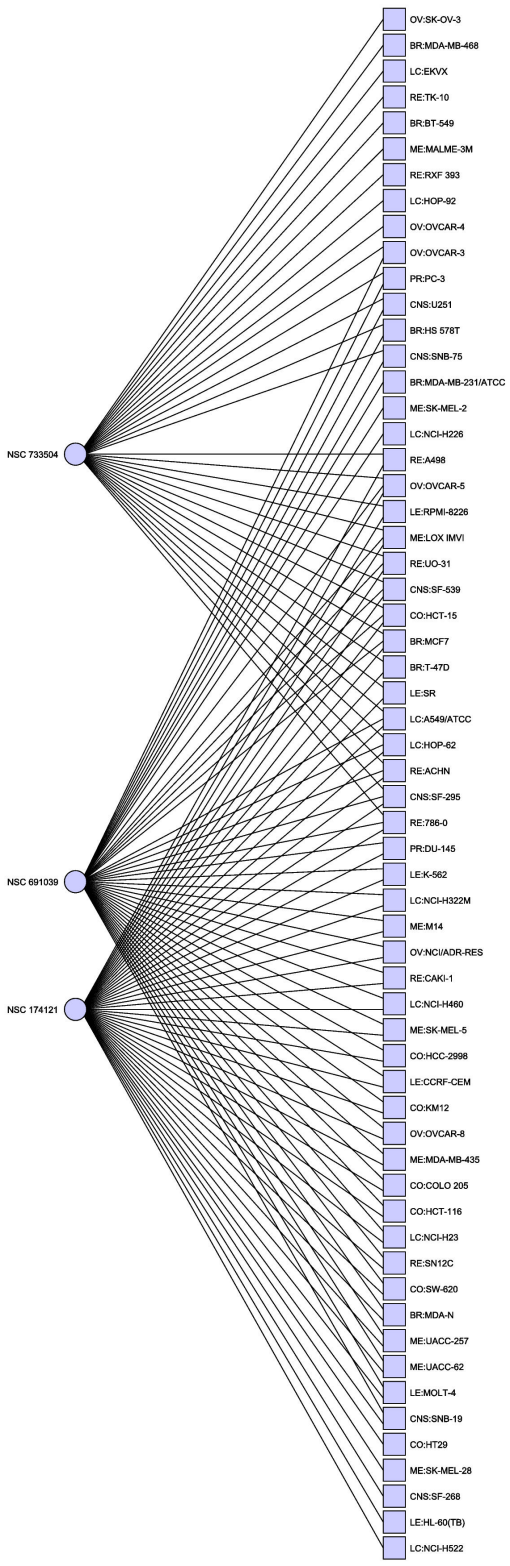
Minimal hitting set hitting for the NCI60 dataset. This hitting set hits all cell lines at least once, but is further optimized to hit all target cell lines the maximal number of times. Of particular note are NSC 174121, a methotrexate derivative and NSC733504, Everolimus/Afinitor, both known anti-cancer agents.

From here we employ more a more recent version of the NCI60 dataset (2009 as compared to Vazquez's 2006). At the time of writing, the latest NCI60 dataset includes 14 additional cell lines, however we remove these, as there is insufficient response data in the dataset, leading to inflated hitting set sizes. The latest data also includes a further 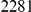 compounds. We note that employing the new GI50 response data we are able to uncover 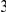 element hitting sets involving compounds not available in the earlier dataset (an example is given in Table 1 and Figure 1), in particular Everolimus (NSC 733504) a drug now used for the treatment of advanced renal cancer which is also giving positive results in phase II trials for metastatic melanoma [24], [25]. However there have recently been some concerns over the provenance of some of the cell lines in the NCI60 dataset. In particular Lorenzi *et al.* [26] suggested that the MDA-N cell line, nominally a breast cancer cell line is in fact similar the M14 and MDA-MB-435 cell lines, and thus should be is in fact a melanoma cell line. Chambers [27] however suggests that although M14 and MDA-MB-435 are identical cell lines, they may not in fact be melanoma cell lines. We do not attempt to resolve this dispute, however with regard to this, and as a indication of the flexibility of the method we employ we consider both the case where MDA-N is a breast cancer cell line and the the case where MDA-N is a melanoma cell line.

**Table 1:** Minimal hitting set using 2009 NCI60 data.

NSC Number	Compound Name
174121	Methotrexate Derivative
691039	(S)-7-Hydroxy-1,2,3-trimethoxy-10-methylsulfanyl-6,7-dihydro-5H-benzo[a]heptalen-9-one
733504	Everolimus/Afinitor

Minimal hitting set for NCI60 GI50 response data from 2009.

**Figure 2:**
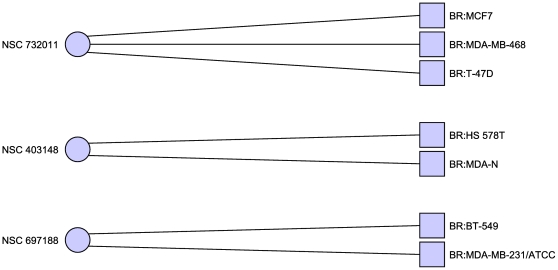
Minimal hitting set hitting only breast cancer cell lines. Including the disputed MDA-N cell line. This hitting set also reveals additional structure with each drug targeting a specific, disjoint subset of the breast cancer cell lines. Only cell lines with at least one adjacent compound are shown.

**Figure 3:**
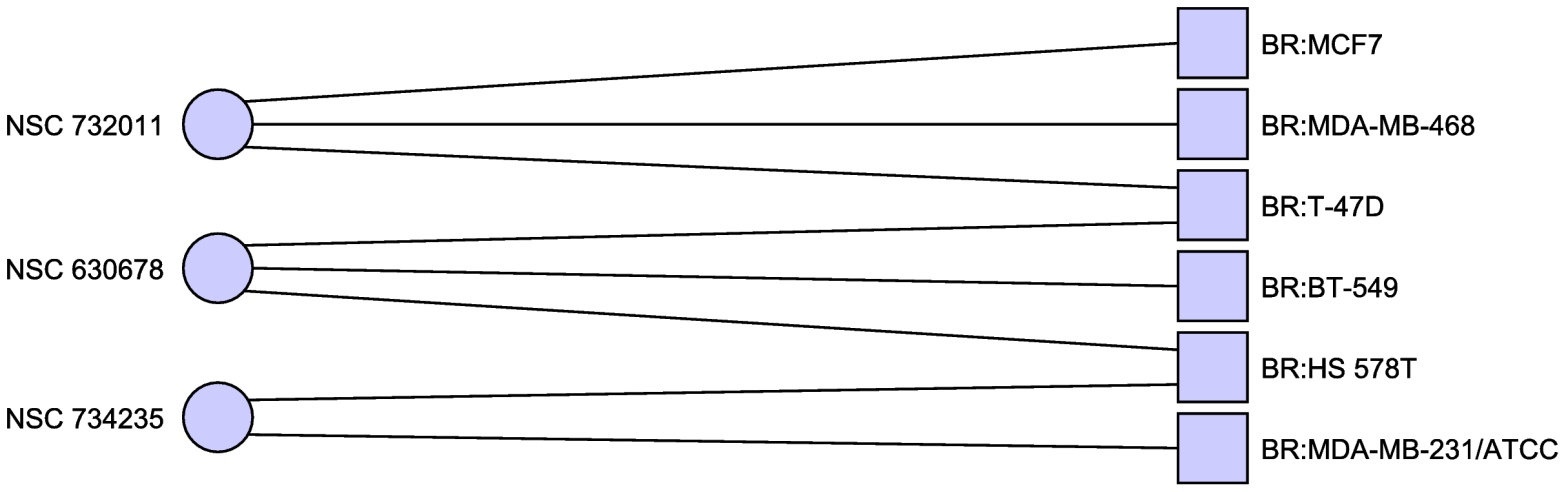
Minimal hitting set hitting only breast cancer cell lines. Excluding the disputed MDA-N cell line. In this case the hitting set is much less clearly separated, though two of the cell lines are now hit twice. Only cell lines with at least one adjacent compound are shown.

**Figure 4:**
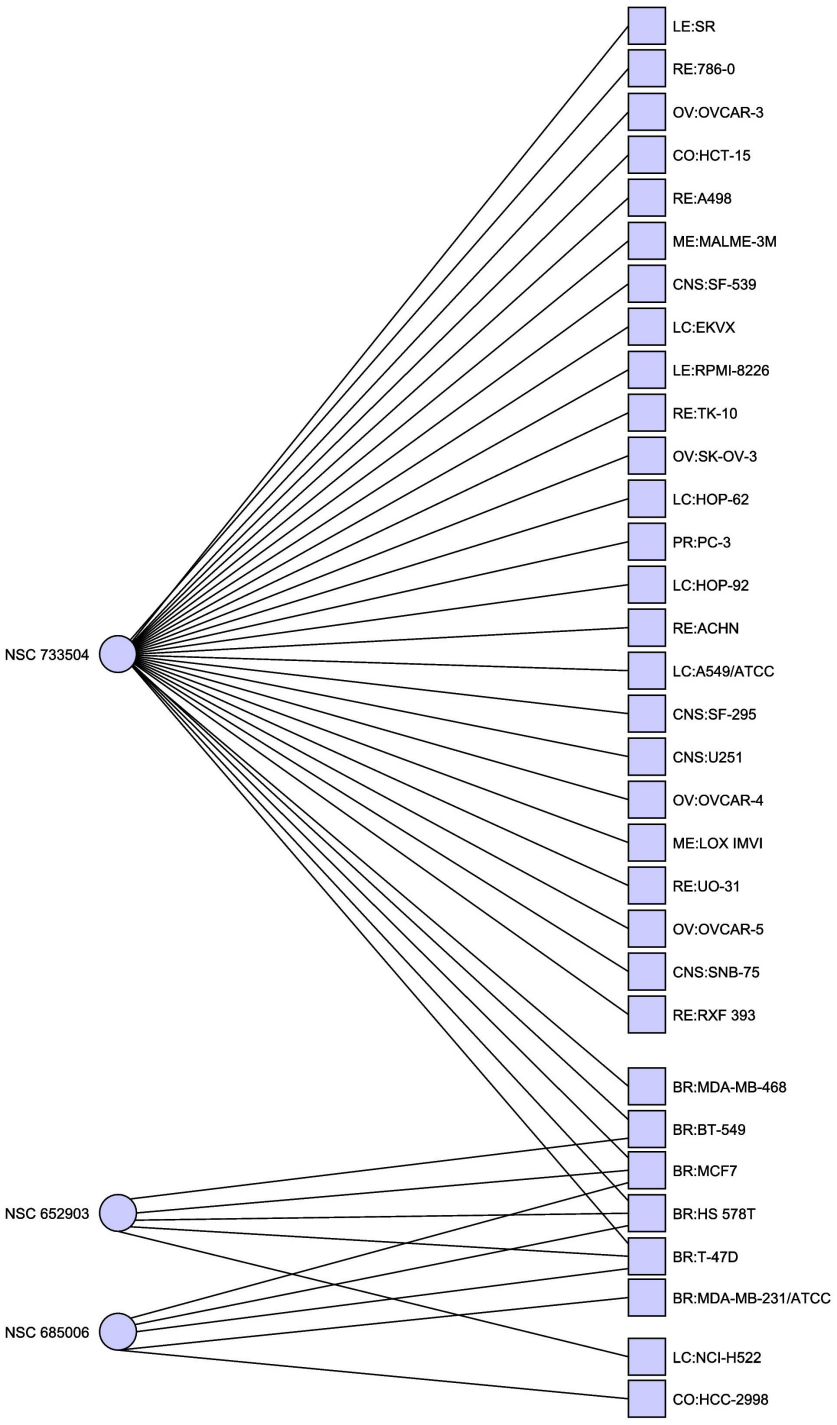
Minimal hitting set hitting only breast cancer cell lines. Excluding the disputed MDA-N cell line. In this case we allow non-breast cancer cell lines to be hit at most once. By relaxing the restriction on hitting non-breast cancer cell lines, we obtain a hitting set which hits more of the breast cancer cell lines repeatedly. The trade-off being that other cell lines are also affected, increasingly the likelihood that non-cancerous cells are also affected by the treatment, as the compounds are less specific to a particular genetic signature. Only cell lines with at least one adjacent compound are shown.

**Figure 5:**
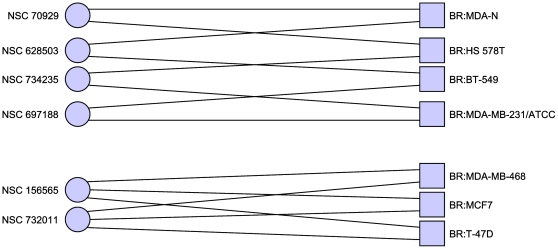
Minimal hitting set hitting breast cancer cell lines twice. Including the disputed MDA-N cell line. In this case the breast cancer cell lines separate neatly into two groups, with the first group forming a cycle and the second group forming a complete bipartite graph. Only cell lines with at least one adjacent compound are shown.

**Figure 6:**
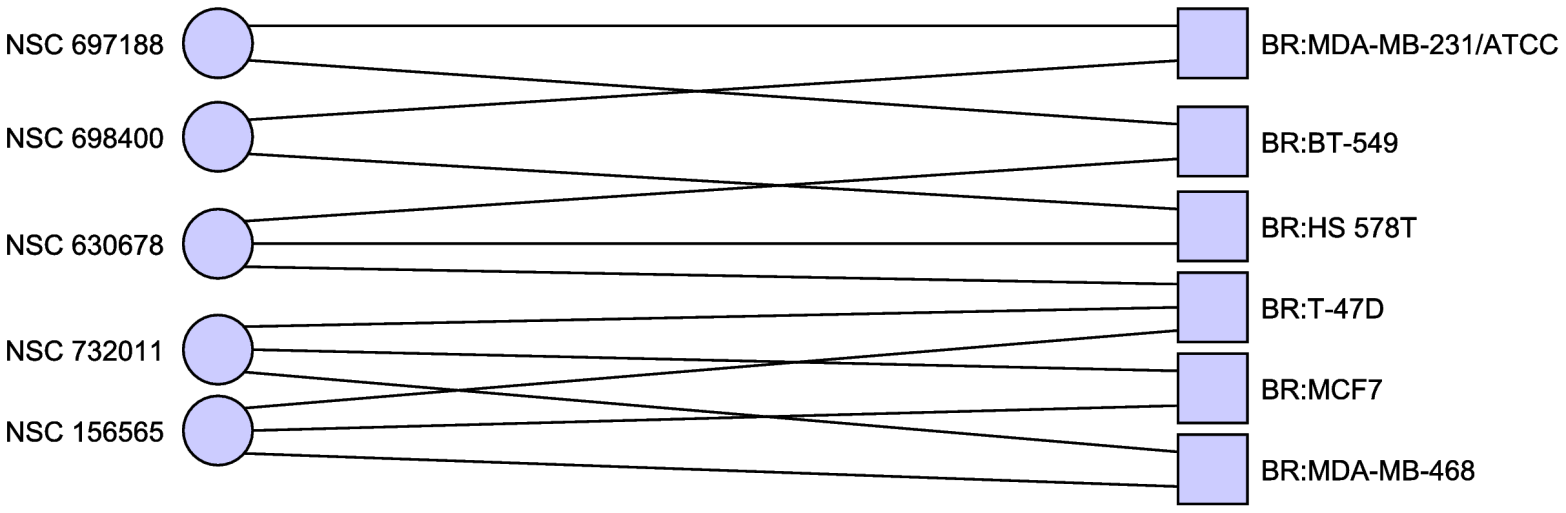
Minimal hitting set hitting breast cancer cell lines twice. Excluding the disputed MDA-N cell line. Without the MDA-N cell line, the breast cancer cell lines do not separate, although the complete bipartite component is a subgraph of this graph, however we gain a greater number of hits per cell line in this case. Only cell lines with at least one adjacent compound are shown.

Employing the (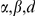)-Hitting Set model gives more flexibility in what kind of therapy we would like to pursue. For instance, by choosing 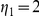 for all vertices, we are able to find a hitting set that hits every cell line at least twice (see Table 2). However the size of this hitting set is 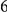, which is likely to be beyond the point where the trade off between anti-cancer efficacy and side effects is acceptable. Fortunately we can exploit (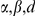)-Hitting Set more intelligently. For example we may wish to find a hitting set that specifically targets breast cancer cell lines – for which we set all breast cancer cell line vertices to have 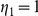 and all other cell lines to have 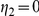. This gives a hitting set that hits *only* breast cancer cell lines, which may be useful in minimizing unwanted peripheral damage to non-breast cancer cells. This gives a hitting set with three elements. In the case where we considered MDA-N to be a breast cancer cell line (see Table 3 and Figure 2) this set includes the compound deoxypodophyllotoxin, which is known to induce apoptosis [28]. If we consider MDA-N as a melanoma cell line we obtain a different hitting set (see Table 4 and Figure 3). If we relax our requirements an allow other cell lines to be hit at most once we can obtain a hitting set that hits the breast cancer cell lines more (Table 5 and Figure 4). The results when we set 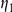 to 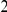 for all breast cancer lines are given in Table 6 and Figure 5 (including MDA-N) and Table 7 and Figure 6 (excluding MDA-N). We note particularly that in the case where MDA-N is included, the optimal hitting set uncovered includes Docetaxel, a well known anti-cancer agent [29] for several cancer types including breast cancer. Interestingly Docetaxel is also currently included in several clinical trials examining its potential as part of a multi-drug therapy [30]–[34].

**Table 2:** Minimal double hitting set.

NSC Number	Compound Name
147340	Anisomycin hydrochloride
174121	Methotrexate derivate
314018	Ansamitocin derivate TN-006
691039	(7S)-7-hydroxy-1,2,3-trimethoxy-10-methylsulfanyl-6, 7-dihydro-5H-benzo[a]heptalen-9-one
712807	Capecitabine
733504	Everolimus/Afinitor

Minimal hitting set hitting each cell line at least twice.

**Table 3:** Minimal hitting set targeting only breast cancer.

NSC Number	Compound Name
403148	Deoxypodophyllotoxin
697188	2-(4-methoxyphenyl)-5-[8-[5-(4-methoxyphenyl)-1,3,4-oxadiazol-2-yl]octyl]-1,3,4-oxadiazole
732011	21-(2-N,N-Diethylaminoethyl)oxy-7.alpha.-methyl-19-norpregna-1,3,5(10)-triene-3-O-sulfamate

Minimal hitting set hitting breast cancer cell lines at least once, and all other cell lines zero times.

**Table 4:** Minimal hitting set targeting only breast cancer without MDA-N.

NSC Number	Compound Name
630678	Streptomyces antibiotic
732011	21-(2-N,N-Diethylaminoethyl)oxy-7.alpha.-methyl-19-norpregna-1,3,5(10)-triene-3-O-sulfamate
734235	isoindolo[1,2-a]quinoxalin-4(5H)-one

Minimal hitting set hitting breast cancer cell lines at least once, and all other cell lines zero times.

**Table 5:** Minimal hitting set targeting breast cancer but allowing other cell lines to be hit.

NSC Number	Compound Name
652903	Saframycin AR1(AH2)
685006	2-imino-8-methoxy-N-phenylchromene-3-carboxamide
733504	Everolimus/Afinitor

Minimal hitting set hitting breast cancer cell lines at least once, and all other cell lines zero times.

**Table 6:** Minimal hitting set hitting breast cancer twice, and no others, with MDA-N.

70929	Hedamycin
156565	1-hydroxy-4-[4-(2-hydroxyethyl)anilino]anthracene-9,10-dione
628503	Docetaxel
697188	2-(4-methoxyphenyl)-5-[8-[5-(4-methoxyphenyl)-1,3,4-oxadiazol-2-yl]octyl]-1,3,4-oxadiazole
732011	21-(2-N,N-Diethylaminoethyl)oxy-7.alpha.-methyl-19-norpregna-1,3,5(10)-triene-3-O-sulfamate
734235	isoindolo[1,2-a]quinoxalin-4(5H)-one

Minimal hitting set hitting breast cancer cell lines at least once, and all other cell lines zero times.

**Table 7:** Minimal hitting set hitting breast cancer twice, and no others, without MDA-N.

156565	1-hydroxy-4-[4-(2-hydroxyethyl)anilino]anthracene-9,10-dione
630678	Streptomyces antibiotic
697188	2-(4-methoxyphenyl)-5-[8-[5-(4-methoxyphenyl)-1,3,4-oxadiazol-2-yl]octyl]-1,3,4-oxadiazole
698400	5-(1,3-benzodioxol-5-yl)-1,2,3,4-tetrahydrobenzo[a]phenanthridine
732011	21-(2-N,N-Diethylaminoethyl)oxy-7.alpha.-methyl-19-norpregna-1,3,5(10)-triene-3-O-sulfamate

Minimal hitting set hitting breast cancer cell lines at least once, and all other cell lines zero times.

**Figure 7:**
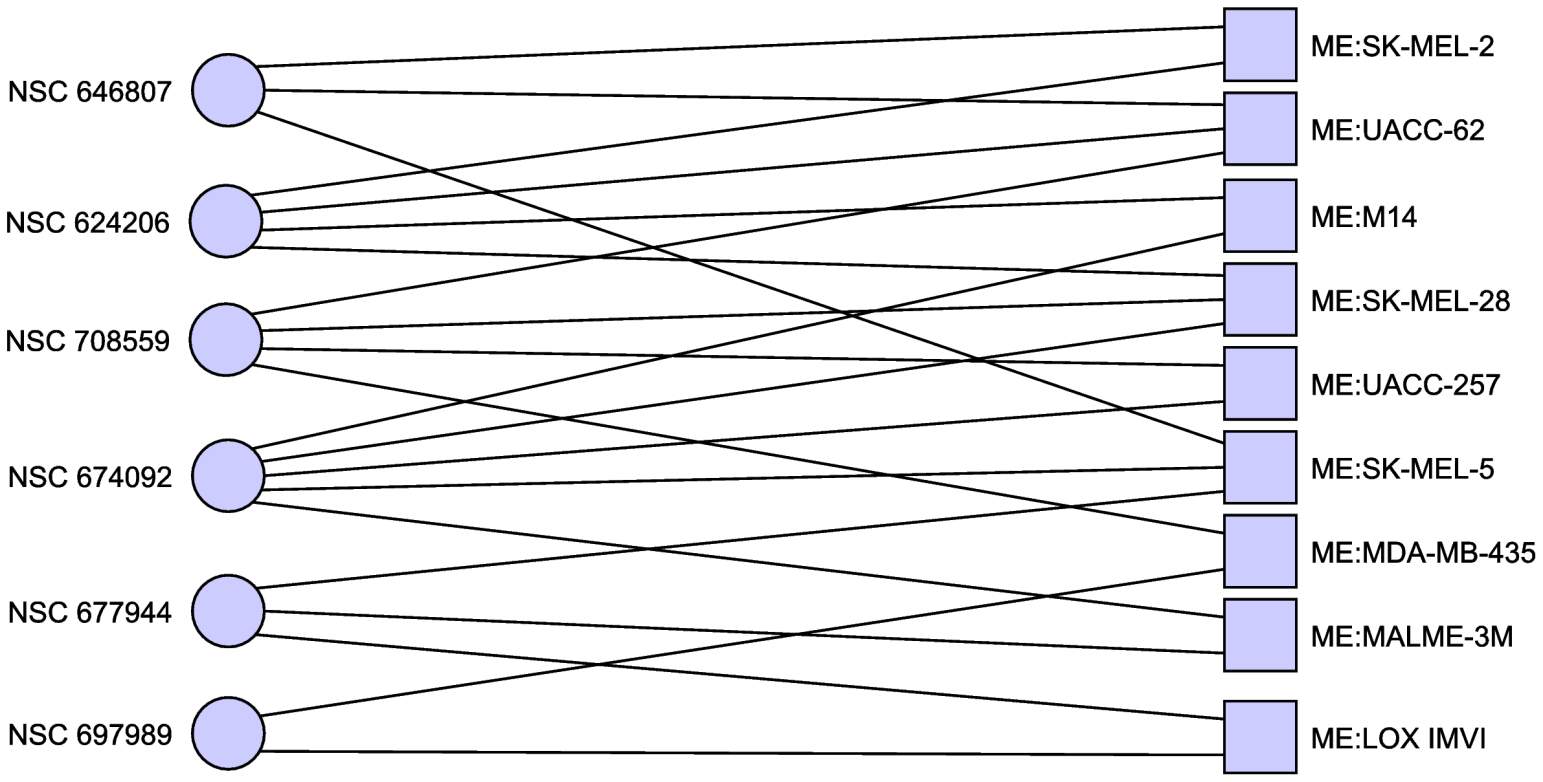
Minimal hitting set hitting melanoma cell lines at least 2 and no other cell lines. This hitting set also maximizes the number of hits on the melanoma cell lines. Only cell lines with at least one adjacent compound are shown.

**Figure 8:**
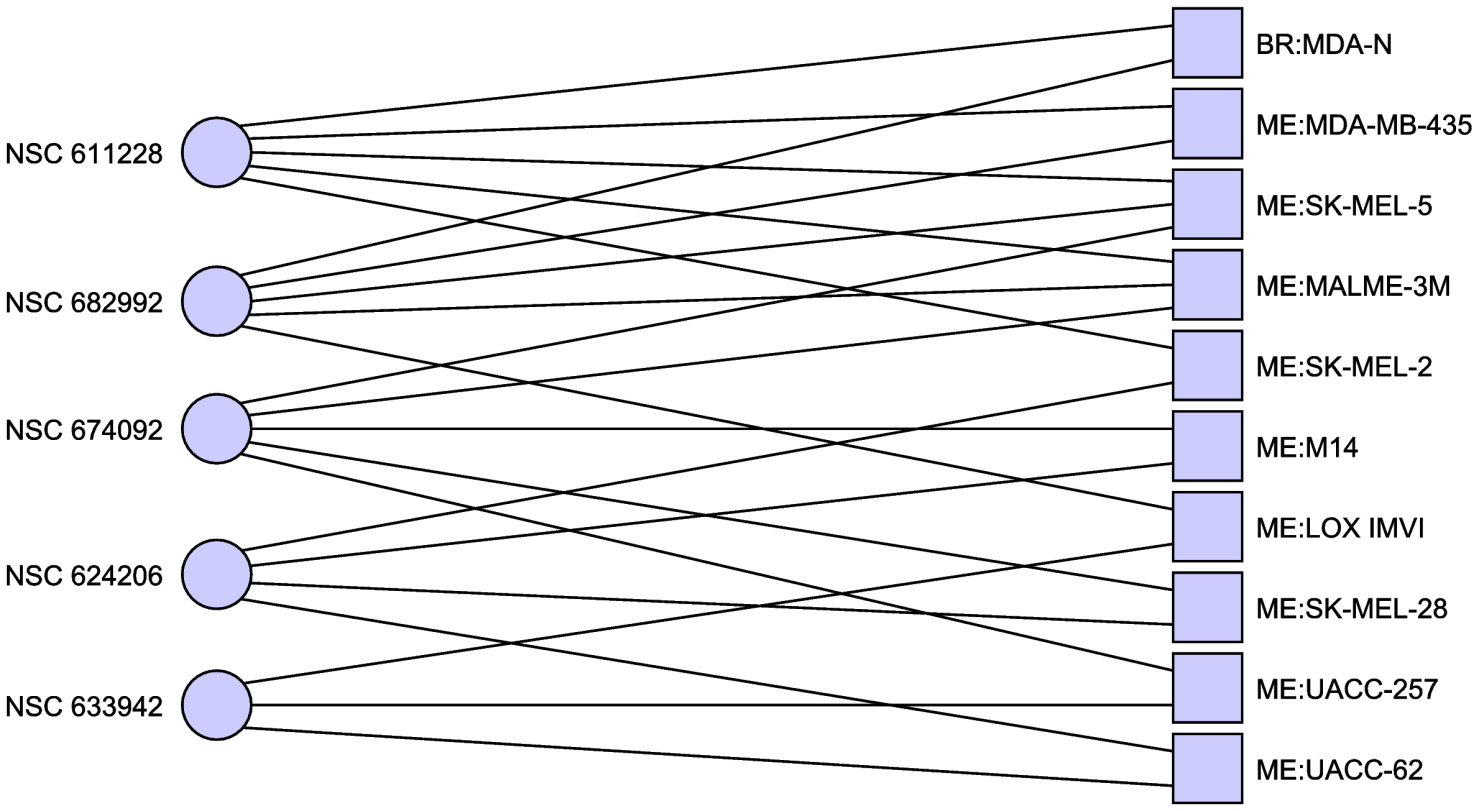
Minimal hitting set hitting melanoma cell lines at least 2 and no other cell lines. Including the disputed MDA-N cell line. It is interesting to note that including MDA-N as a melanoma cell line rather than a breast cancer cell line reduces the size of the minimal hitting set from 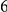 to 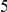. This hitting set also maximizes the number of hits on the melanoma cell lines. Only cell lines with at least one adjacent compound are shown.

**Figure 9:**
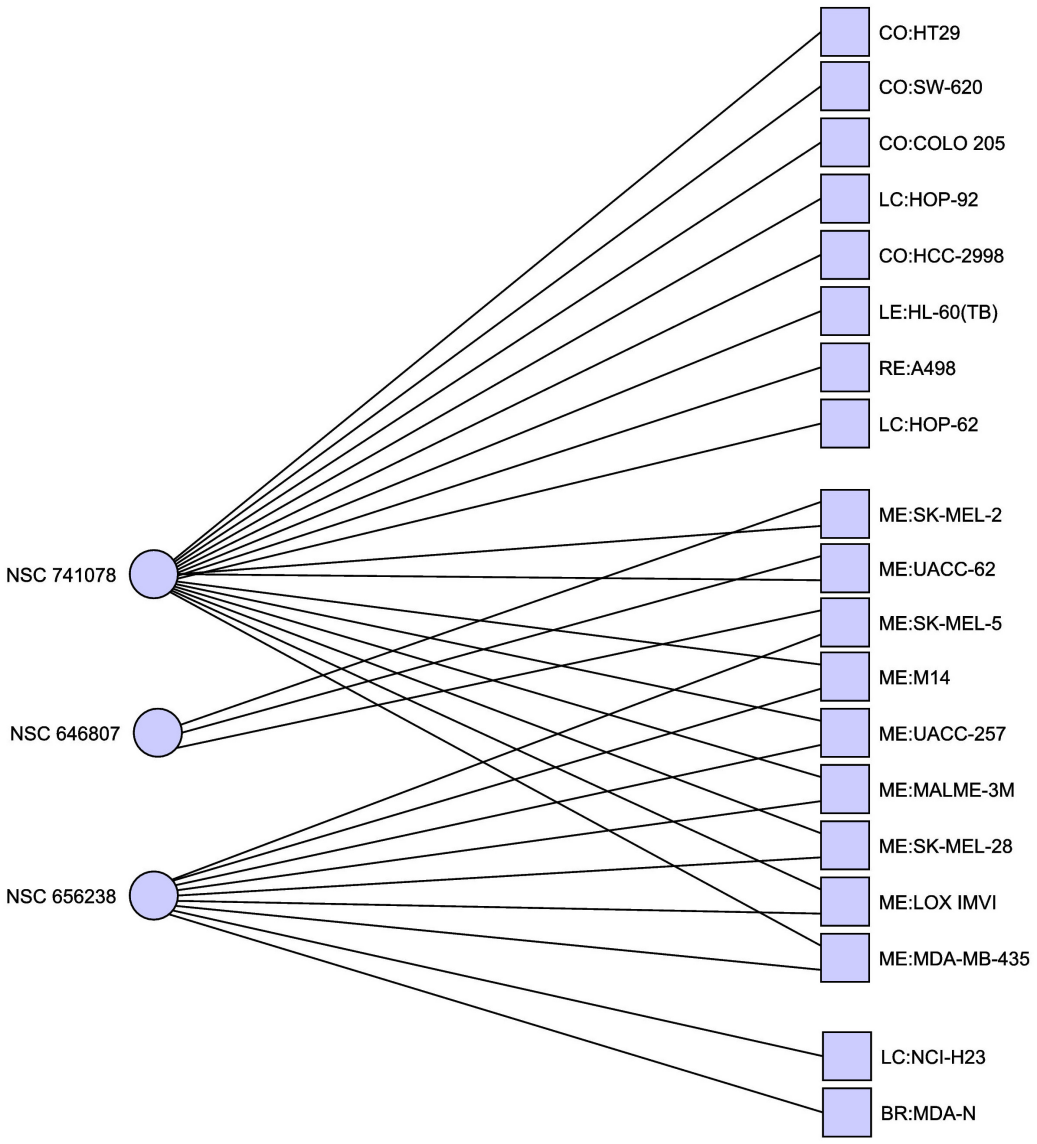
Minimal hitting set hitting melanoma cell lines at least 2 and all other cell lines at most once. For this we consider MDA-N as a non-melanoma cell line, however it is also hit by the hitting set, though only once. This hitting set also maximizes the number of hits on the melanoma cell lines. Only cell lines with at least one adjacent compound are shown.

**Figure 10:**
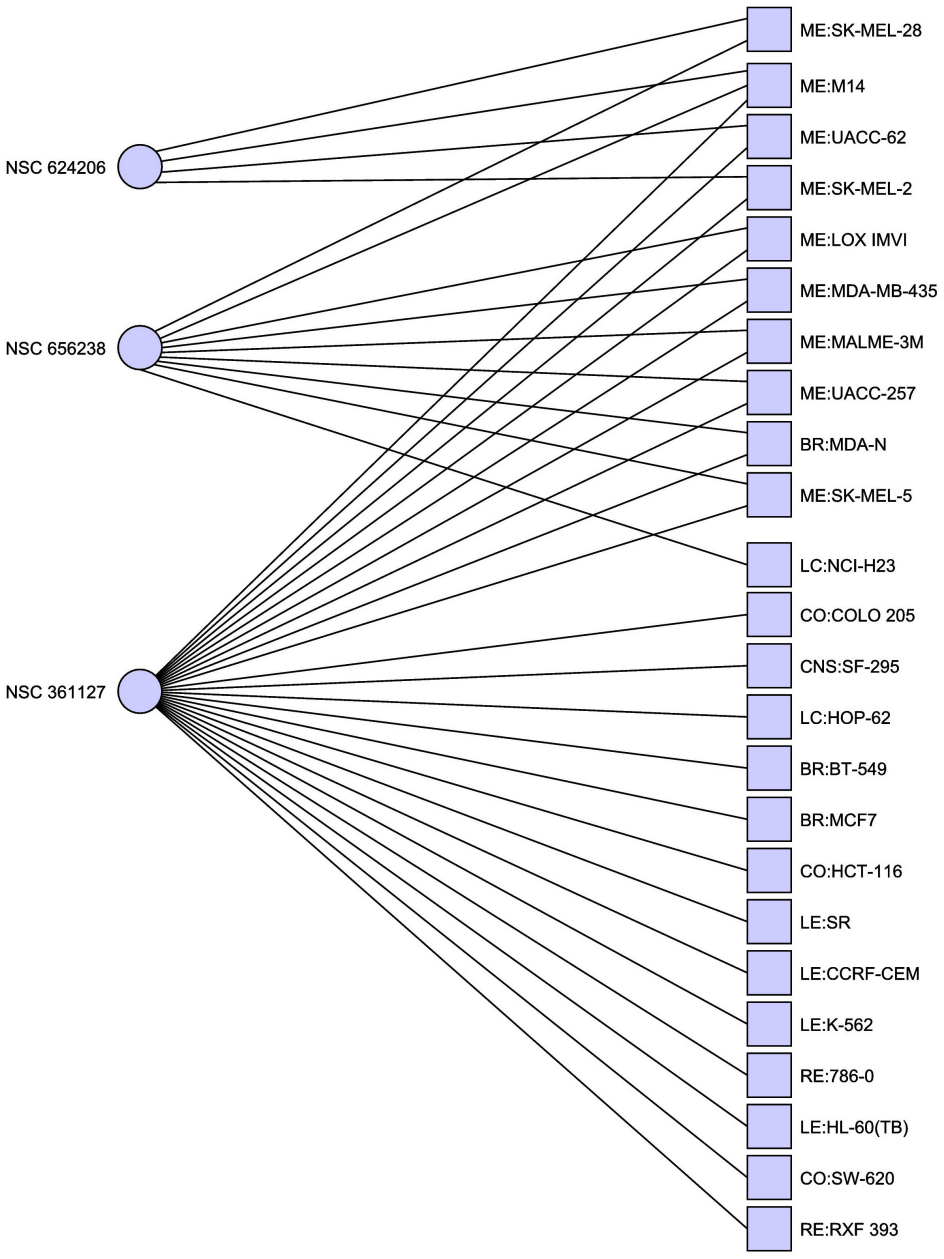
Minimal hitting set hitting melanoma cell lines at least 2 and all other cell lines at most once. Including MDA-N as a melanoma cell line. The key difference with the case where we consider MDA-N to be a non-melanoma cell line is that in this case we obtain a hitting set that hits the melanoma cell lines slightly more. Only cell lines with at least one adjacent compound are shown.

In another example, we may wish to target melanoma cell lines exclusively, and furthermore, we may wish to attack each cell line with at least two drugs at once. However in this case (where 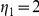 for melanoma cell lines and 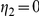 for all others) the minimal hitting set size is 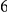 (or 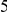 if MDA-N is included as a melanoma cell line – Table 8 and Figures 7 & 8). Considering that a therapeutic cocktail involving 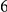 compounds may have excessive side effects, we can relax the requirements, and allow 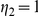 for non-melanoma cell lines. In this case we find that the smallest hitting set is of size 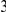. By altering the focus when solving the kernel by fixing the hitting set size (
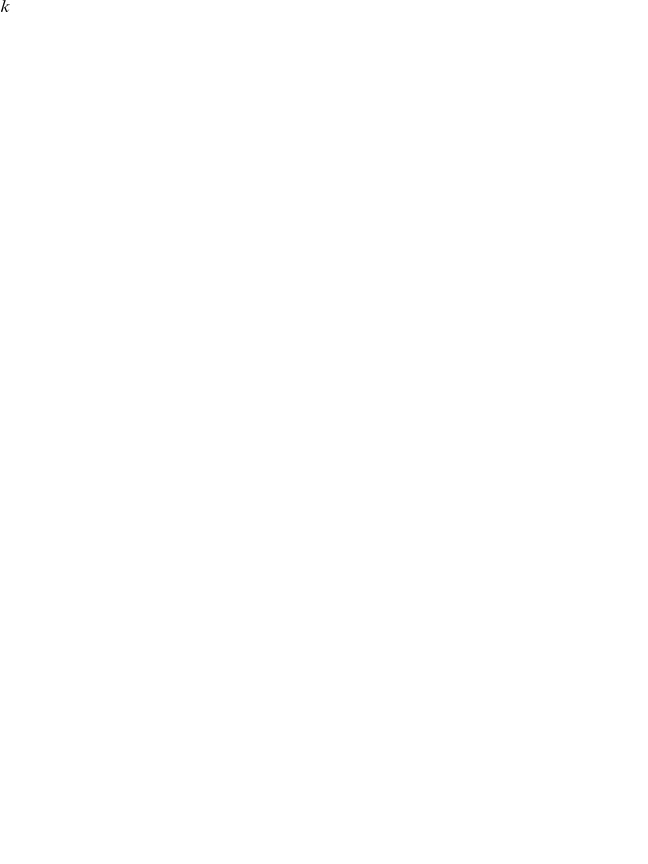
) at 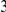 and maximizing the total degree of the vertices in the hitting set, subject to the 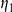 and 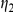 constraints, we can obtain the minimal size hitting set that hits our targets as much as possible, within the bounds given by the constraints. This results in the hitting sets in Tables 9 & 10 and Figures 9 & 10. Of note is AZD6244, which is currently involved in 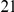 anti-cancer drug trials [35] and has been identified as a potent kinase inhibitor [36], [37].

**Table 8:** Minimal hitting set targeting melanoma twice, without MDA-N.

624206	N-[2-[(4-chlorophenyl)methyldisulfanyl]ethyl]decan-1-amine hydrochloride
646807	2-(2-Isonicotinoylhydrazino)-N-(3-methyl-1,4-dioxo-1,4-dihydro-2-naphthalenyl)-2-oxoacetamide
674092	2-phenyl-N-[3-[4-[3-[(2-phenylquinoline-4-carbonyl)amino]propyl]piperazin-1-yl]propyl]quinoline-4-carboxamide hydrochloride
677944	6-[2-(4-hydroxy-3-methoxyphenyl)ethylamino]quinoline-5,8-dione
697989	dicopper 2-acetyloxy-3,5-di(propan-2-yl)benzoate
708559	2-(3,4-dichlorophenyl)-N-methyl-N-[3-[methyl(3-pyrrolidin-1-ylpropyl)amino]propyl]acetamide

Minimal hitting set hitting melanoma cell lines at least twice and no others. This result does not include MDA-N as a melanoma cell line.

**Table 9:** Minimal hitting set targeting melanoma, without MDA-N.

NSC Number	Compound Name
646807	2-(2-Isonicotinoylhydrazino)-N-(3-methyl-1,4-dioxo-1,4-dihydro-2-naphthalenyl)-2-oxoacetamide
656238	2-Methyl-4,8-dihydrobenzo[1,2-b:5,4-b′]dithiophene-4,8-dione
741078	AZD6244 (ARRY-142886)

Minimal hitting set hitting melanoma cell lines at least twice, all others at most once, maximizing the degree of the melanoma cell line vertices.

**Table 10:** Minimal hitting set targeting melanoma, with MDA-N.

NSC Number	Compound Name
361127	Destruxin E
624206	N-[2-[(4-chlorophenyl)methyldisulfanyl]ethyl]decan-1-amine hydrochloride
656238	2-Methyl-4,8-dihydrobenzo[1,2-b:5,4-b′]dithiophene-4,8-dione

Minimal hitting set hitting melanoma cell lines at least twice, all others at most once, maximizing the degree of the melanoma cell line vertices.

### Conclusion

Given the size of modern datasets, and the expectation that they will only get larger, it is clear that we require efficient approaches to solving important computational biology problems. The first phase of any such approach is simply defining the problem at hand. Unfortunately once clearly stated, many such problems are 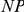-hard or worse. However this need not mean that we must resort to inexact or approximate approaches, which could be undesirable in a field such as drug selection. Parameterized Complexity provides a toolkit for dealing with nominally hard problems, and identifying cases where despite super-polynomial running times, we may still expect good performance.

The drug selection problem as examined here is one such problem. It is modeled well by the 
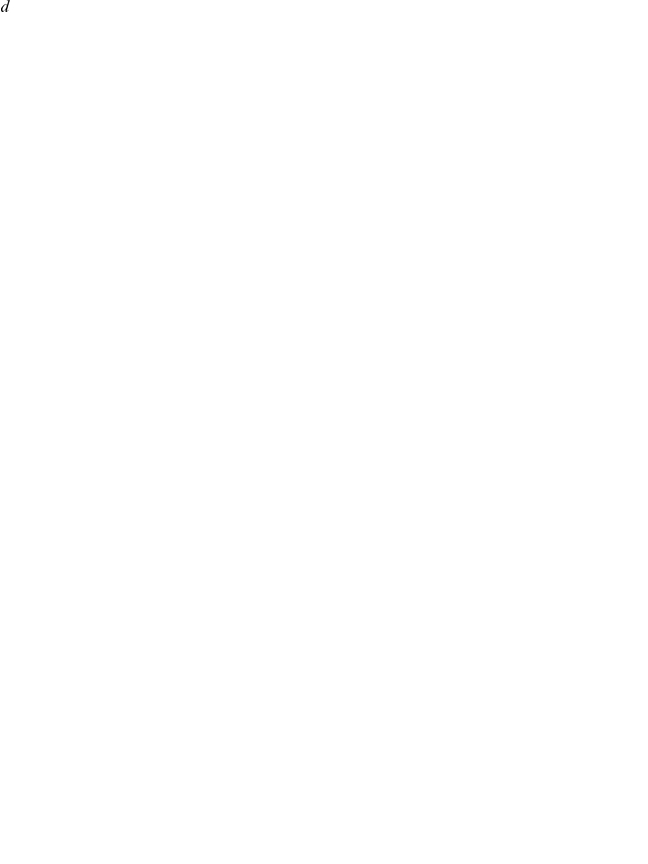
-Hitting Set problem, which is fixed-parameter tractable when parameterized by the maximum size of the hitting set. Therefore we can expect that despite being 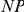-complete, it would be relatively quick to solve when these parameters are small. However we demonstrate that the much more flexible variant (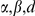)-Hitting Set is also fixed-parameter tractable, with only the addition of a single parameter - the maximum of the minimum number of times any vertex should be hit. With (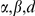)-Hitting Set we are able to better control the nature of the hitting set uncovered, and thus tailor any such hitting set to a useful set of constraints, such as limits on which cell lines are to be hit, the maximum any of these can be hit and of course the minimum number of times any cell line should be hit. Moreover we can solve this problem quickly, and guarantee optimality - without any notable restrictions on the parameters and constants. This allows the quick generation of possible drug combinations for testing, with guarantees of a certain baseline performance, eliminating the need to exhaustively test all possible combinations, which would be financially and temporally prohibitive.

In brief this paper provides a robust and flexible methodology for multiple drug selection, which can easily be applied to other domains that are modeled by the 
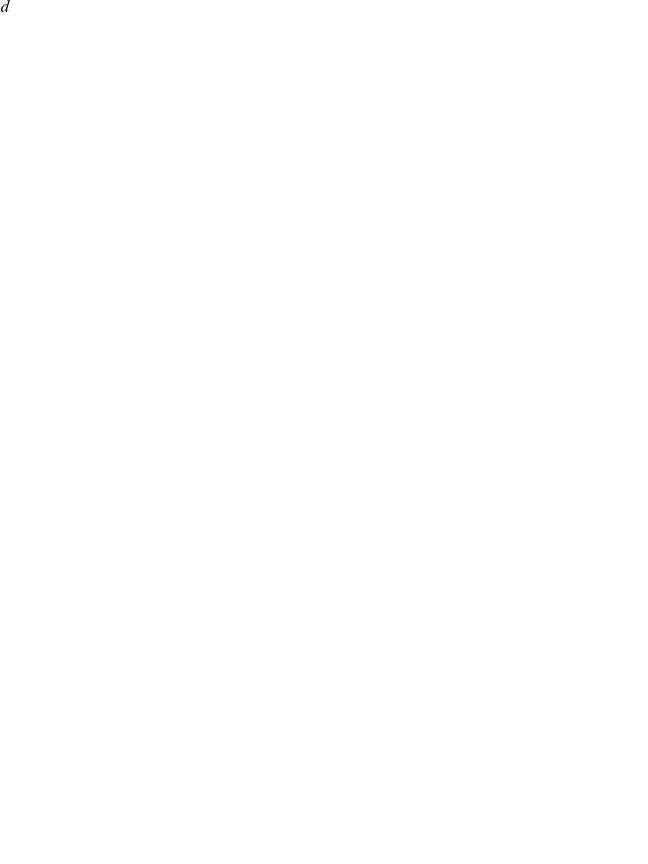
-Hitting Set problem, with a sound theoretical background as to why and how the problem can be solved efficiently, despite its 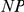-completeness. Moreover the existence of a kernelization for (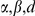)-Hitting Set indicates that even without using a specialized commercial solver such as CPLEX, the problem is readily scalable to large datasets. Given the speed at which we are able to solve instances with on the order of 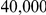 vertices, we can expect that much larger datasets are also solvable in a reasonable time.

A future extension that may be of interest would be to somehow encode in the problem the notion that some hitting vertices are incompatible, e.g., two compound may have severe adverse interactions, and thus can never be used together as a therapy, regardless of their individual usefulness.

## Materials and Methods

### Dataset and Computational Method

The dataset primarily employed is the NCI60 DTP Human Tumor Cell Line Screen, available from [22]. We use the version released in October 2009, and downloaded in April 2010. The raw dataset is presented as a series of cell line and compound pairs, along with the GI50 response measurement (the method for producing the measurements is also detailed by [22]) for that pair plus concentration information and statistical information. Where there are multiple entries for the same compound-cell line pair, we select the entry resulting from the experiment using the highest concentration of the compound. We extract this data into a matrix cross indexed by the NSC number of the compound and the name of the cell line. Where an entry does not exist for a given compound-cell line pair, we enter “NA” for that entry in the matrix.

Once the data is in this matrix format we threshold the data according to the method used by Vazquez [4] whereby the raw data is subject to a z-transformation over a logarithmic scale and then any value above a certain threshold expressed in terms of the standard deviation to 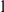, and anything below, including “NA” values, to 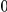. In line with Vazquez we choose two standard deviations as our particular threshold for this paper, though this is adjustable.

We then construct a graph for the hitting set instance using the Java Universal Network/Graph Framework (JUNG) [38] with the SetHypergraph class, representing each compound with a vertex and each cell line with a (hyper)edge which carries a weight indicating the number of times that edge is to be hit. This graph is then reduced to remove vertices of zero degree, edges with no incident vertices (which are noted as technically this would indicate a no instance unless that edge does not require hitting) and vertices that are only adjacent to edges that require zero hits. This basic reduction alone typically reduces the number of vertices significantly, bringing the graph within a reasonable size for immediate processing. From a theoretical standpoint the constant 
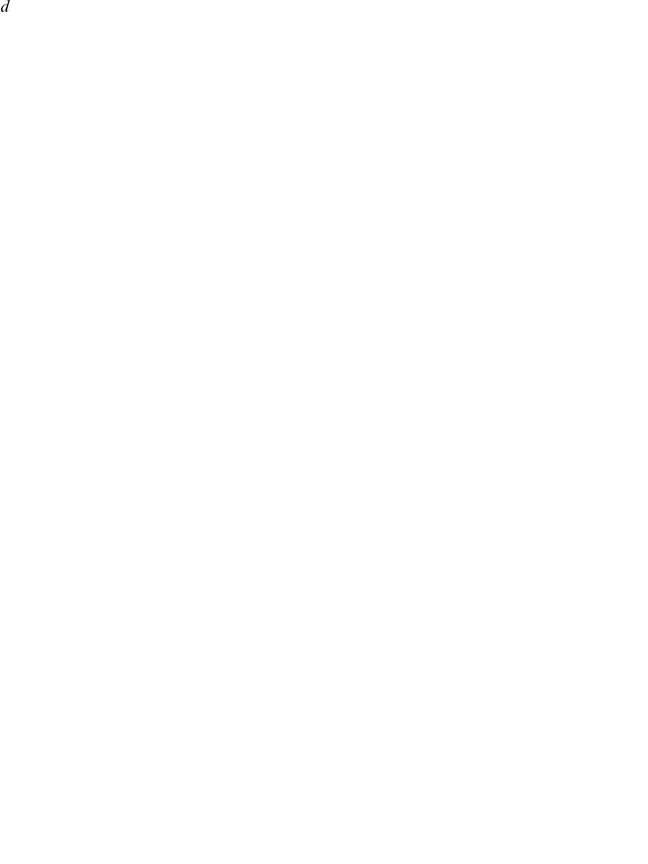
 is of importance, for the graph constructed as stated, 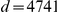 (as we allow the natural value, rather than imposing an external limit). In practice a 
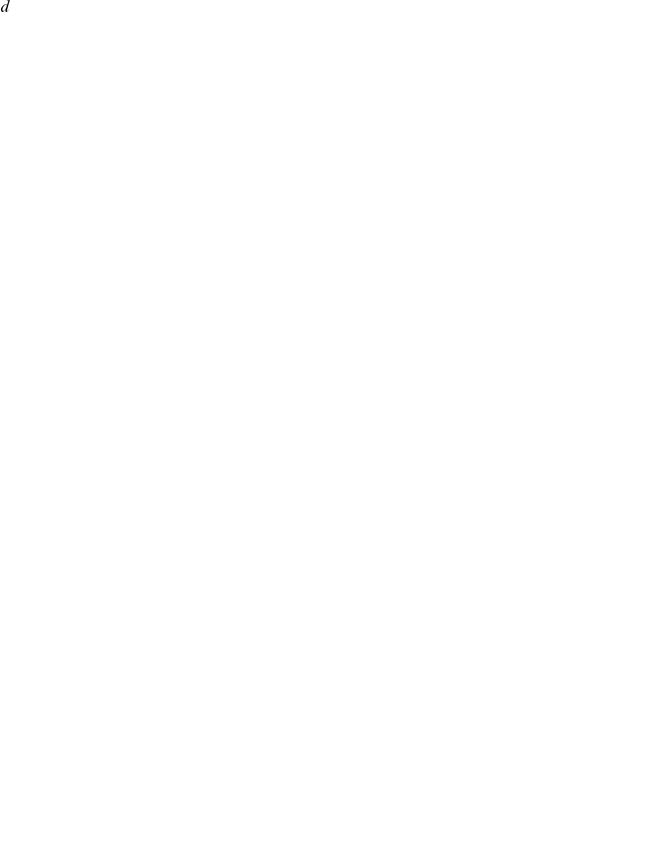
 value of this magnitude proves perfectly workable, and returning to the theoretical viewpoint indicates that the instance is in a sense already kernelized.

Once the graph is reduced, we construct an integer programming instance equivalent of the problem given the graph, and pass this instance to CPLEX [23] (version 11.200) and search for an optimal solution to one of two objective functions, given the constraints of the number of hits for each cell line (given by the 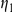 value). The first objective function simply minimizes the size of the hitting set (
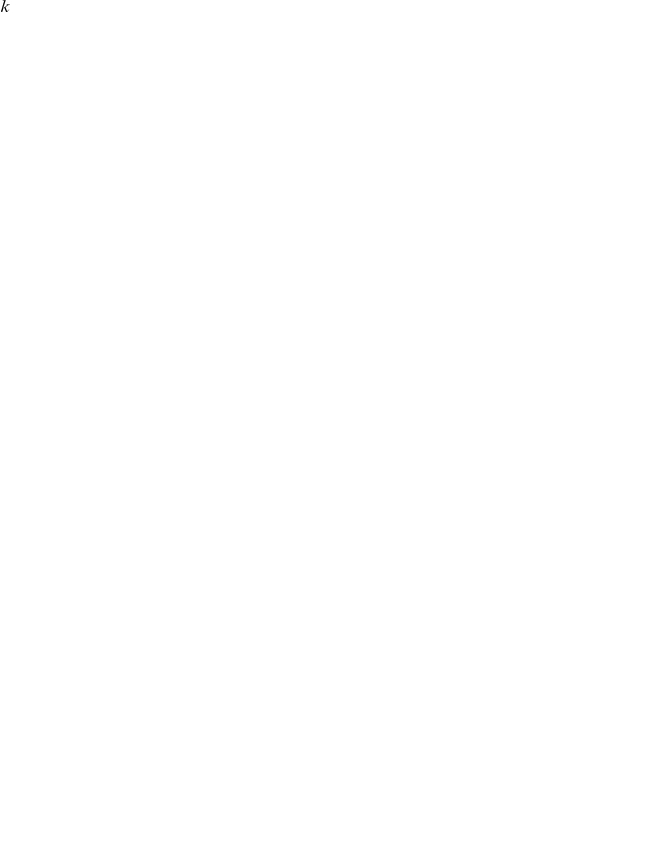
), for the second objective function we fix the size of the hitting set, and maximize the number of hits on vertices where no maximum number of hits has been set (the 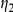 value). As part of this search CPLEX may apply some unspecified proprietary reduction process.

The figures were created using yEd Graph Editor [39].

The computer hardware employed is a Dell PowerEdge III Dual Xeon 5550 server with 32Gb of RAM, operating Red Hat Linux 64 bit EL 4 Server.

### Theoretical Background and Kernelization Proof

#### Graph Theory and Notation

A *(simple undirected) graph* consists of a set 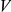 (the vertices), and a set 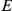 of two element subsets of 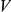 (the edges). A *bipartite graph* is a graph where the vertices are partitioned into two partite sets, where all edges have one endpoint in one set and the other endpoint in the other set, i.e., 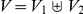 and 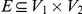.

Given a graph 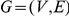 and two vertices 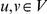, we denote the edge between 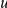 and 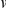 by 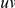 or equivalently 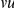. Given two vertices 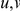 in 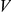, if there is an edge 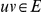 we say that 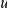 and 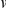 are *adjacent* and the 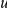 and 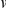 are *incident* on 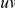. Given a vertex 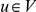, the set 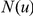 is the *(open) neighborhood* of 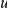 and consists off all vertices adjacent to 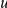 in 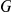, we extend this notion in the natural way to sets of vertices.

#### Parameterized Complexity

A *parameterized (decision) problem* is a formally defined computational problem consisting of three components; the input, a special part of the input called the parameter, and the question. Following Flum and Grohe's [40] definition we may assume that the parameter is derived from a polynomial time computable mapping from the input to the natural numbers. A parameterized problem 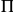 is *fixed-parameter tractable* if there is an algorithm 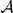 such that for every instance 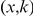 where 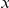 is the input, 
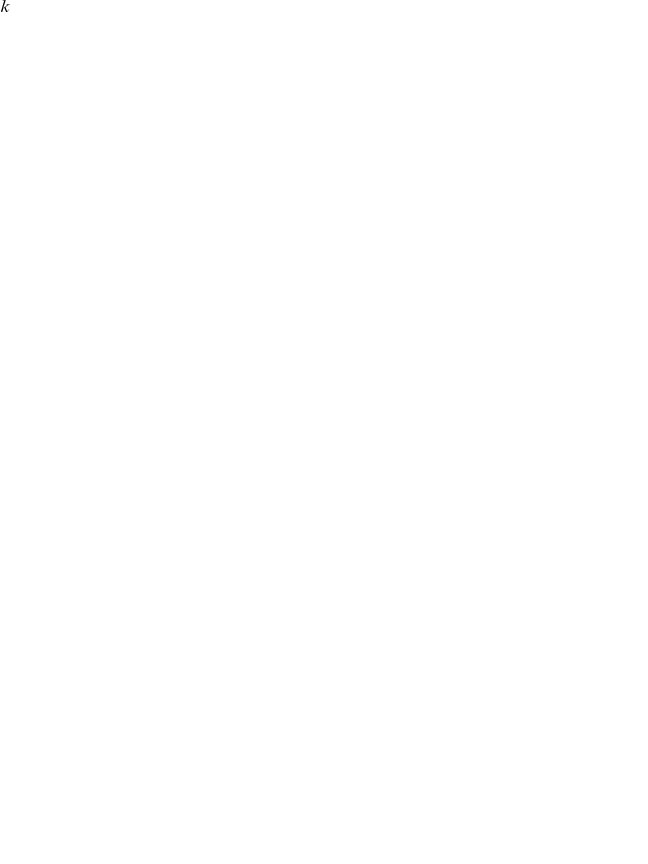
 is the parameter and 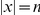, 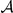 correctly answers Yes or No in time bounded by 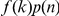 where 
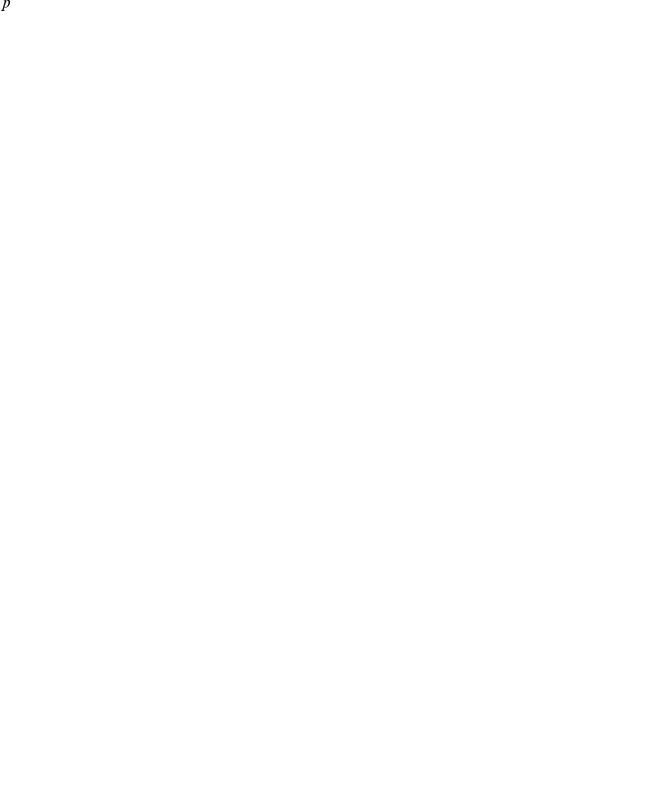
 is a polynomial and 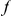 is a computable function.

A *polynomial time kernelization* (or just *kernelization*) is a polynomial time mapping that given an instance 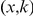 of a parameterized problem produces a new instance 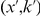 of the problem such that:

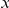
 is a Yes-instance if and only if 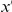 is a Yes-instance,

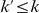
 and

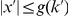
 for some computable function 
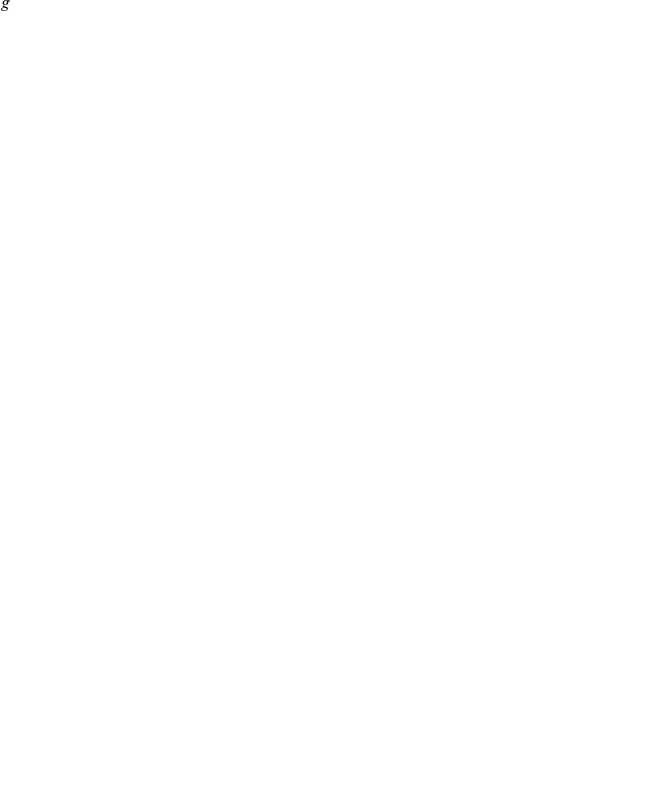
.

It is easy to see that if a problem has kernelization, then it is fixed-parameter tractable. It is also easy to prove that if a problem is fixed-parameter tractable, then it has a kernelization [41].

Parameterized complexity has a fully developed theory for determining when a problem is unlikely to be fixed-parameter tractable, but as this is not necessary for this work, we refer the reader to the monographs of Flum and Grohe [40] and Downey and Fellows [42] for full discussion, and simply state that if a problem is 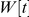-hard or 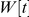-complete for any 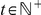, then the problem is not fixed-parameter tractable unless certain complexity theoretic assumptions are false, which seems unlikely.

### The Fixed-Parameter Tractability of (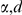)-Hitting Set

Our kernelization for (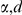)-Hitting Set follows the basic format of Abu-Khzam's kernelization for 
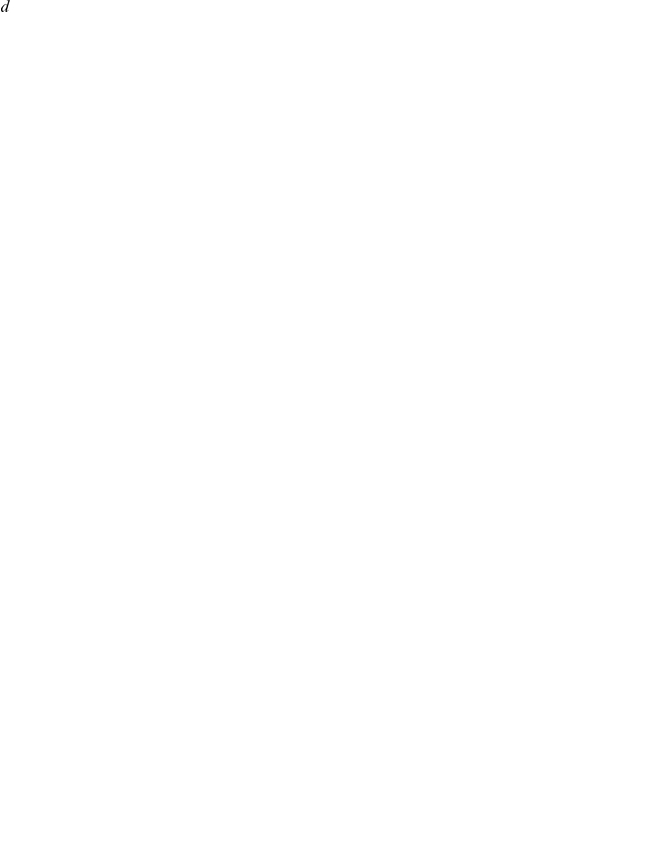
-Hitting Set [18].

Let 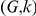 be an instance of (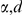)-Hitting Set which we assume to have been preprocessed for nonsense input such as vertices 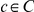 with 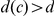 or 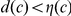. Therefore we may assume that for all 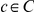 we have 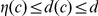 and that for all vertices 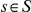 we have 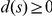.

We first apply Reduction Rules 1 to 3 exhaustively, before applying Rules 4 and 5.:

**Reduction Rule 1:** If there is a vertex 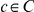 with 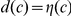 then for every vertex 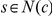 for every vertex 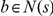 reduce 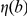 by 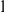, delete 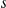 from 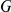 and reduce 
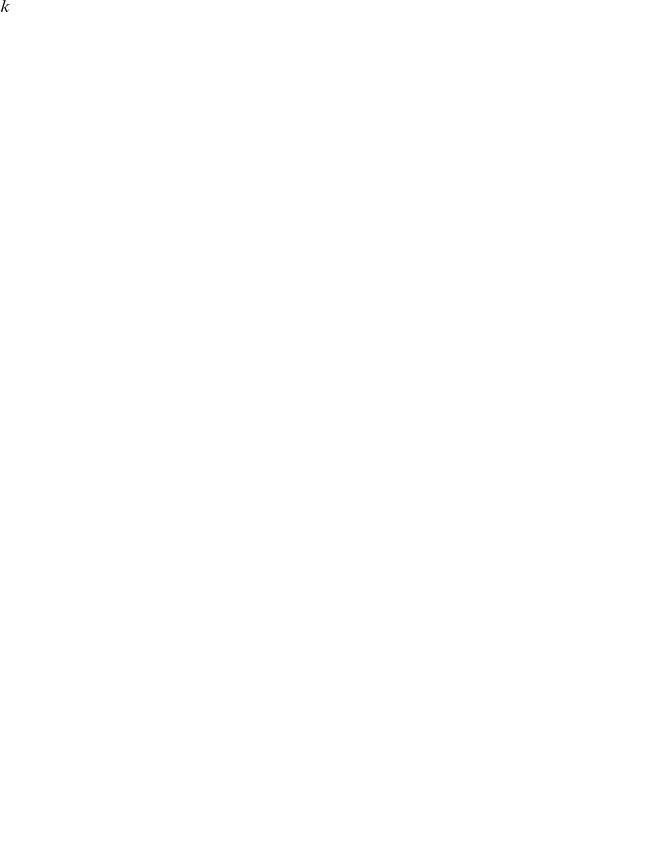
 by 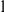. Finally, delete 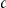 from 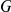.

**Lemma 1** *Reduction Rule 1 is sound*.

*Proof*. If such a vertex 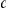 exists, then all its neighbors in 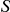 must be in the hitting set, and we can remove them from the graph after suitably noting the effect for the vertices of 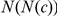.

Note in particular that this rule effectively allows us to assume that 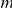 is at most 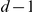. This will be used implicitly in Reduction Rule 4.

**Reduction Rule 2:** If there is a vertex 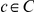 with 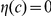, delete 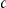 from 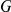.

**Lemma 2** *Reduction Rule 2 is sound*.

*Proof*. Clearly 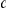 requires no vertices to hit it, so may be ignored.

**Reduction Rule 3:** If there are two vertices 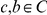 such that 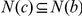 and 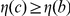, delete 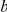 from 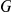.

**Lemma 3** *Reduction Rule 3 is sound*.

*Proof*. If two such vertices 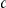 and 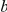 exist, then any hitting set that hits 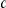 at least 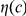 times will hit 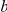 at least 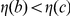 times.

Let 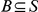 be a set of size 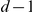 vertices such that 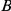 is the pairwise intersection of the neighborhoods of a vertex set 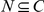. Let 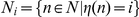.

**Reduction Rule 4:** Let 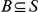 and 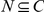 be vertex sets as described. For each 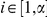 such that 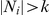 add a vertex 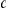 to 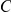 with 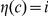 and edges such that 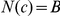 and delete 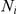 from 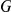.

**Lemma 4** *Reduction Rule 4 is sound*.

*Proof*. Let 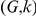 be a Yes-instance of (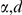)-Hitting Set. Then there is a set 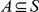 with 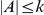 that hits each element 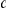 of 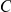 at least 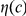 times. Assume that there are sets 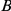 and 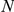 as described in the reduction rule and that for some 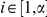 we have that 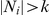. Let 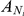 be the subset of 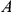 that hits 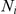. Assume further that 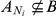, then for each 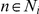 there is at least one other vertex in 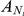, but then 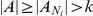, which contradicts the assumption that 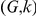 is a Yes-instance.

Therefore the set 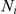 must be hit by 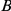, so we may restrict our search to the intersection.

**Lemma 5** *Reduction Rule 4 can be computed in polynomial time*.

*Proof*. Given a set of vertices 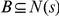 for some 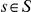 with 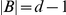, we construct an auxiliary graph 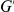 by taking for each 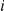 the subgraph of 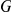 induced by the vertices 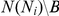. If there is a maximum matching in 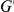 of size greater than 
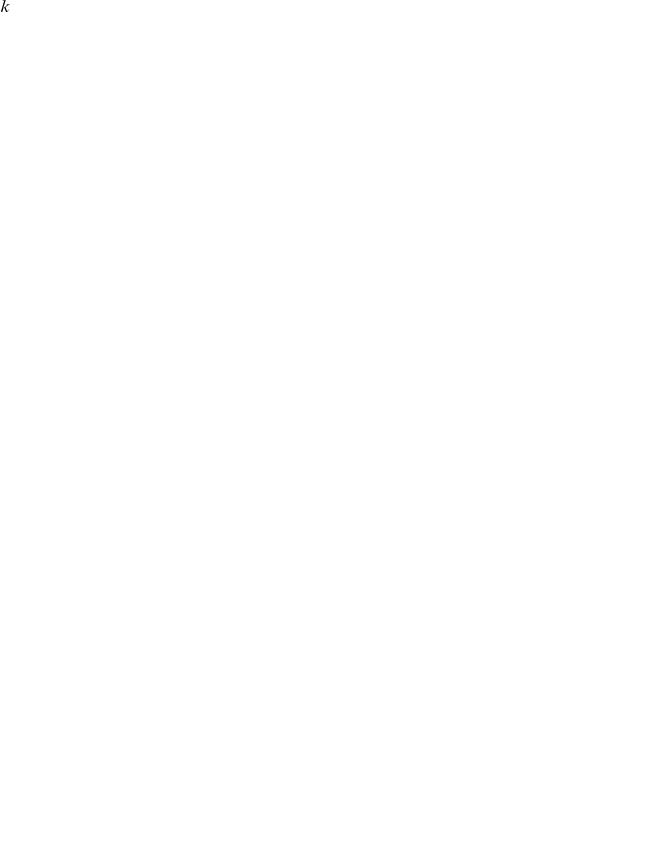
, then the matched vertices from 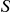 form the required set with pairwise neighbohood intersection 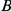.

As 
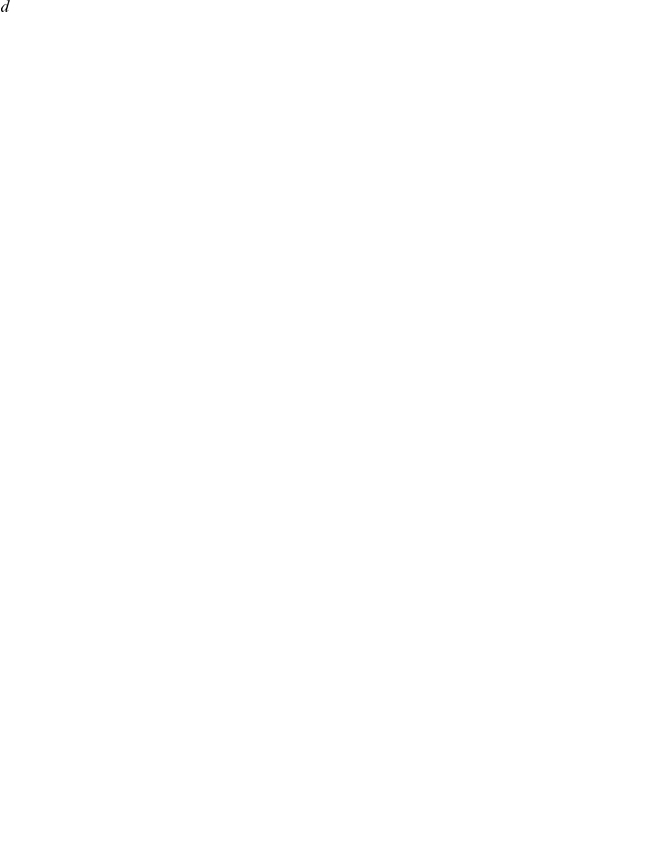
 is a constant, we can iterate over all sets of vertices of size 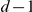 in time 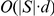. The matchings can be computed in time 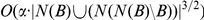.

**Definition 6 (Weakly Related Vertices)** Given two vertices 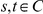, 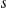 and 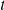 are *weakly related* if 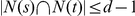, and both 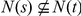 and 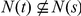.

Let 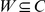 be a maximal set of pairwise weakly related vertices. Let 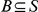 be a set of vertices, and denote by 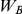 the set of vertices of 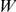 whose neighborhood is a superset of 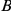. Further denote by 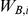 the subset of 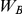 where for each 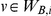 we have 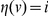.

**Reduction Rule 5:** Compute a maximal collection 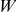 of pairwise weakly related vertices. If 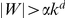 apply the following algorithm:

**for** 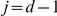 **downto** 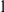 **do**

 **for** 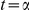 **downto** 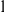 **do**

  **for** each set 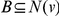 where 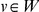 and 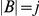 **do**

   **if** 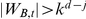 **then**

    Add a vertex 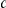 to 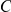, edges such that 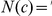 and set 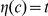.

    Delete 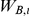 from 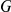.

**Lemma 7** *Reduction Rule 5 is sound*.

*Proof*. We defer the proof of the bound on the size of 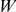 until the proof of Lemma 8.

Let 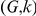 be a Yes-instance of (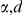)-Hitting Set. Then there is a set 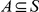 that hits 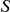 sufficiently. For sets of size 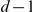, Reduction Rule 4 proves the soundness of the first iteration of the outer loop.

For each other iteration, assume that the iteration for sets of size 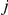 holds, then let 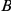 be set of size 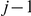 where 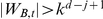 for some 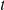. If 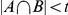 then by the pigeon hole principle there is some vertex 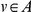 that is in at least 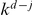 neighborhoods of vertices in 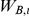, but then 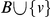 is a set that is the intersection of at least 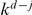 neighborhoods of vertices in some subset of 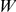, contradicting the correctness of the previous iteration. Therefore the entire set of vertices hitting each 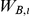 vertex is contained within 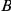 if 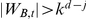, so we may replace 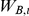 with a single vertex.

Note also that for each element of 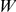 there is at most 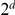 sets 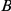, so we may iterate through all sets in time 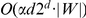, so we can perform the replacements in polynomial time.

**Lemma 8** *If* 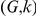 *is a* Yes-*instance of* (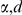)-Hitting Set, *reduced under Reduction Rules 1 to 5*, *then* 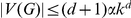.

*Proof*. If 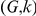 is a Yes-instance of (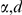)-Hitting Set, then there is a set 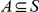 such that for every 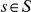 we have 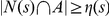 with 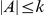.

**Claim 9** 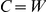.

By construction, every vertex in 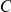 with degree at most 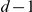 is in 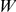. Assume there is some 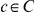 with 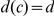 and 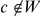, then there must be some vertex 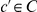 such that 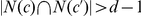, but then as the degree of any vertex in 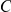 is at most 
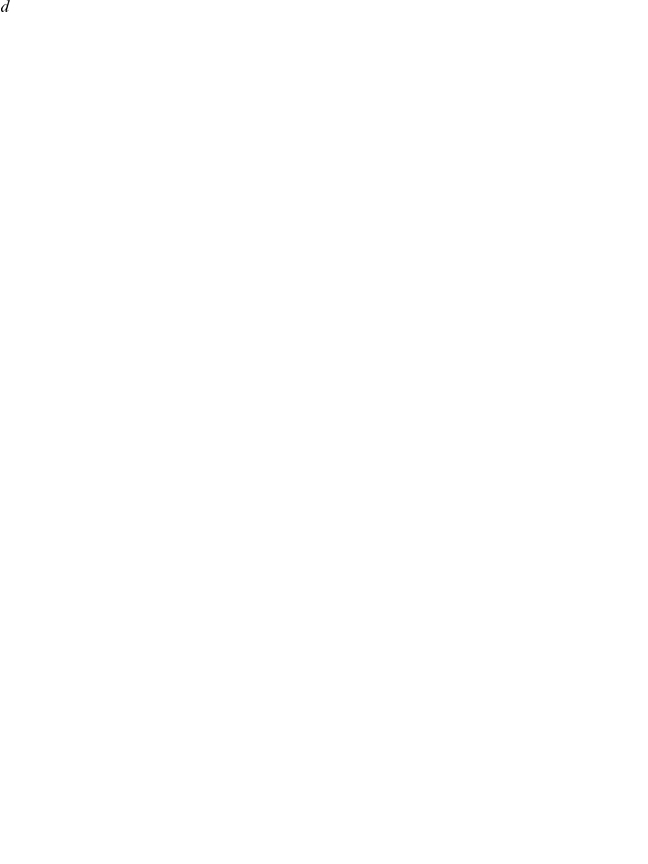
, 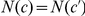, and Reduction Rule 3 would apply. Therefore there are no vertices from 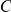 not in 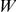.

**Claim 10** 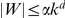.

As 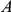 hits each vertex of 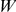 at least once, by Reduction Rule 5 each element of 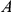 as a singleton is in the neighborhood of at most 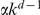 vertices from 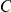. Therefore 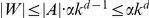.

Combining Claims 9 and 10 we have 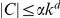. As each vertex of 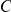 has degree at most 
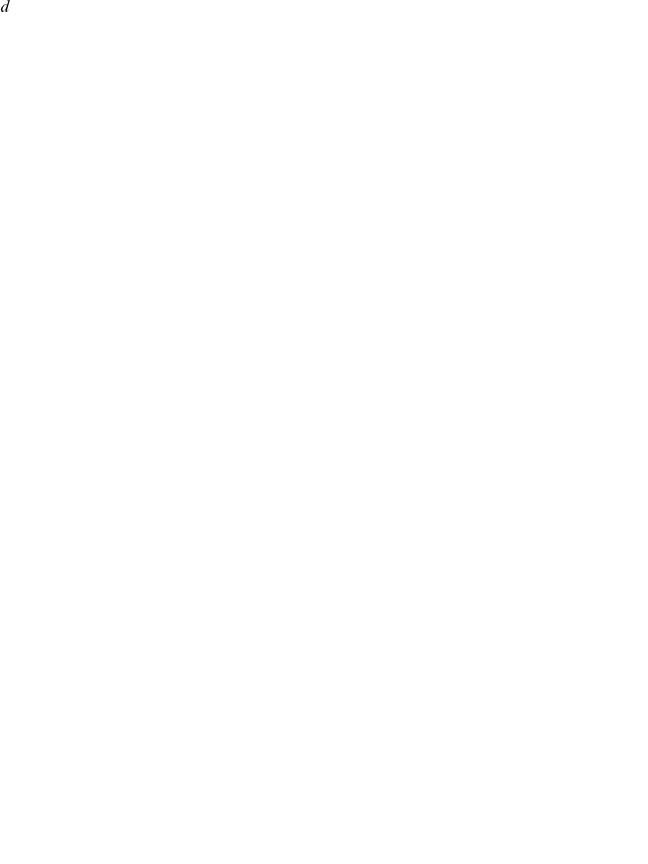
, there are at most 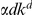 vertices in 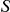, and the bound follows.

**Theorem 11** (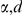)-Hitting Set *is fixed-parameter tractable with parameter* 
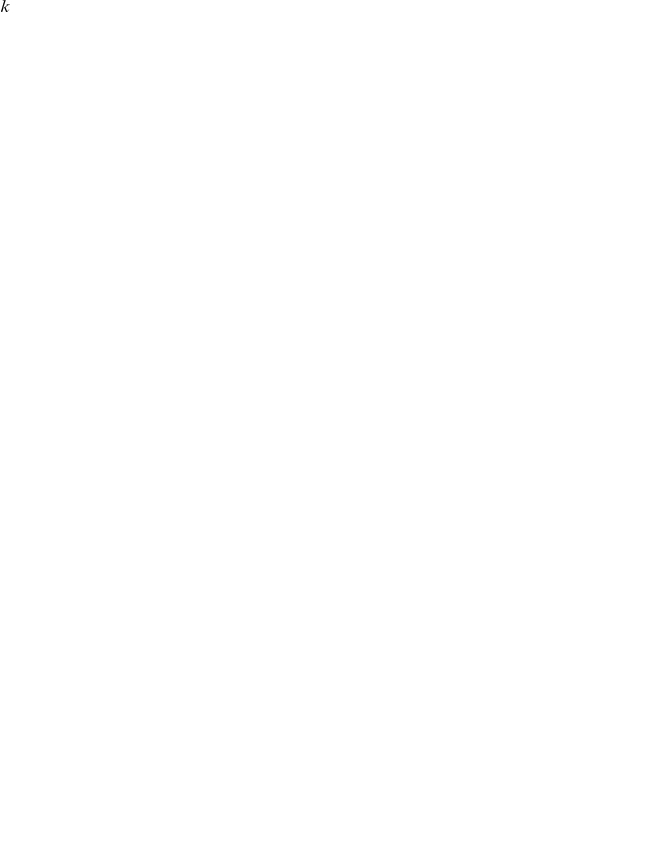
 *and has a kernel of size at most* 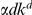.

We note that although 
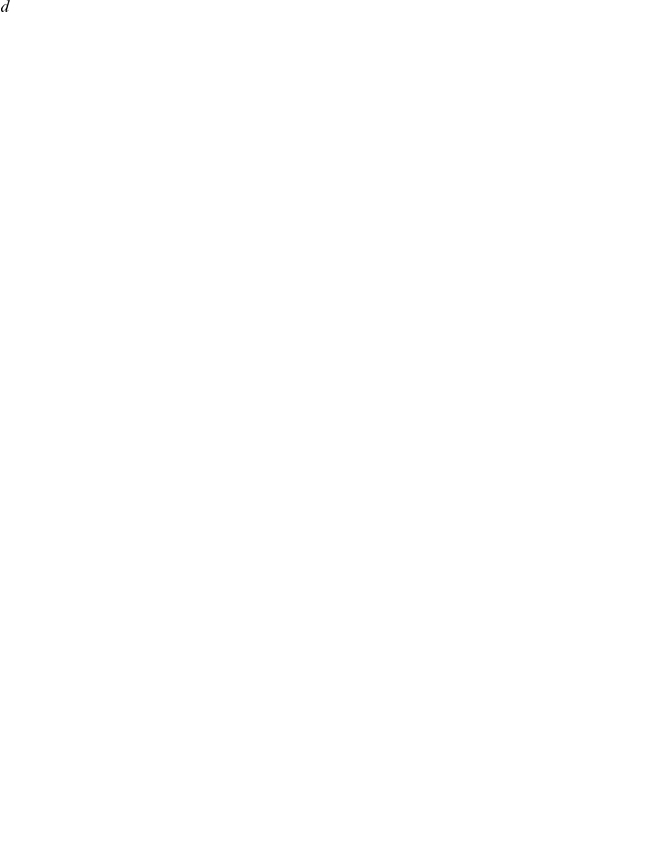
 must be a constant to obtain a polynomial time kernelization, 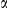 may be alternatively given as an additional parameter, without change to the kernelization.

This kernelization may be extended to an even more general version of the problem, where we not only specify lower bounds for the number of hits, but also upper bounds:

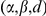
-Hitting Set

*Instance:* A bipartite graph 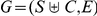 where for all 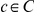 we have 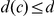, two hitting functions 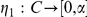 and 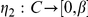 and an integer 
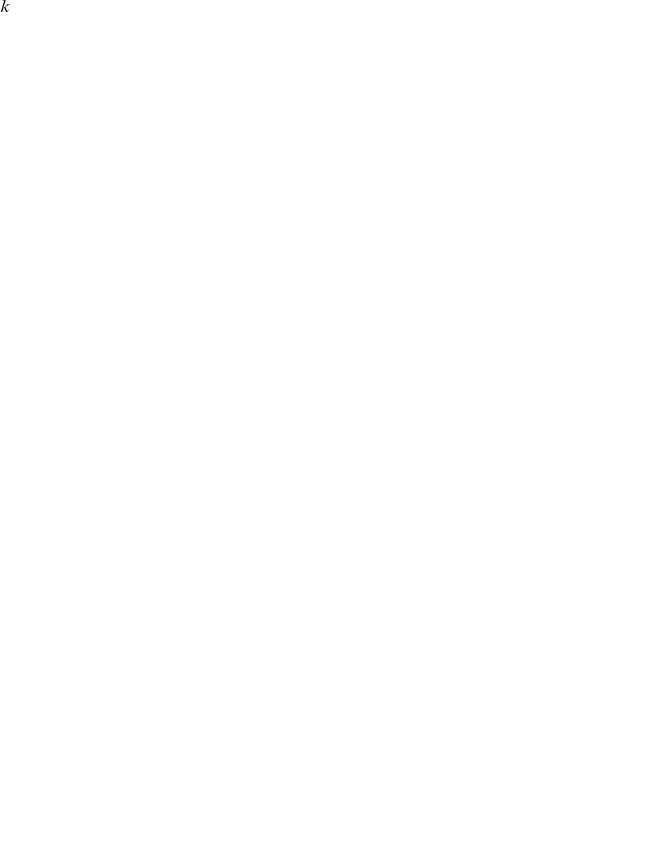
.

*Question:* Is there a set 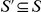 with 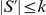 such that for every 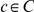 we have 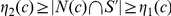
?

**Corollary 12** (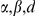)-Hitting Set *is fixed-parameter tractable with parameter* 
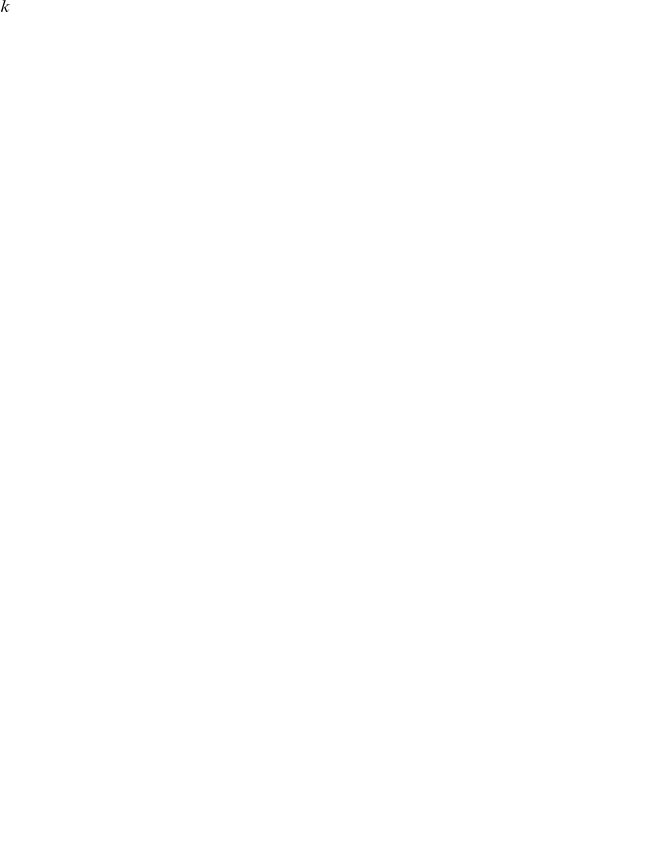
 *and has a kernel of size at most* 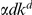.
